# A standard-curve-free ΔΔCt method for rapid GMO compliance screening relative to labeling thresholds

**DOI:** 10.3389/fpls.2026.1918379

**Published:** 2026-08-27

**Authors:** Mei Dong, Tingting Wen, Lixia Meng, Chaohua Miao, Weixiao Liu, Jihong Zhang, Yusong Wan, Wujun Jin

**Affiliations:** Inspection and Testing Center for Ecological and Environmental Risk Assessment of Plant and Plant-Related Microorganisms of the Ministry of Agriculture and Rural Affairs, Biotechnology Research Institute, Chinese Academy of Agricultural Sciences, Beijing, China

**Keywords:** GMO labeling thresholds, GMO quantification, inter-laboratory collaborative verification, quality control samples, ΔΔCt method

## Abstract

Regulatory thresholds for genetically modified organism (GMO) content are widely adopted worldwide for labeling purposes. Routine inspection primarily requires determining whether GMO levels exceed, fall below, or approach the threshold, rather than requiring precise quantification. Derived from the comparative Ct method, the ΔΔCt method enables rapid GMO screening using threshold-matched quality control (QC) samples. In this study, we established a universal ΔΔCt cutoff of ±0.4 as a decision criterion for categorizing samples into three groups relative to the labeling limit. The robustness of this criterion was validated through an inter-laboratory collaborative study involving eight testing laboratories, two GM crop matrices (soybean and maize), and a broad range of GMO concentrations. This method eliminates standard curve preparation while supporting both rapid threshold-based screening and semi-quantitative estimation of GMO content. This dual functionality makes it particularly suitable for high-throughput regulatory testing. Overall, this cost-effective and technically robust workflow provides a practical tool to facilitate the implementation of GMO labeling regulations.

## 1 Introduction

Currently, the industrialization of genetically modified organisms (GMOs) is advancing rapidly, with GMO products play an increasingly prominent role in international agricultural trade and food markets (Agroinvestor, 2025; Kumar et al., 2020; Brookes, 2022). On the other hand, a series of reports on biosafety has sparked significant public concern (Goodman, 2024; Koch et al., 2025). Due to multiple considerations—including international politics, economics, and public acceptance—a growing number of countries have begun to implement labeling requirements for GMO products (Davison and Bertheau, 2008; Garcia-Alonso et al., 2025; Gruere et al., 2009; Lewi et al., 2025; Ryan et al., 2024).

The European Union pioneered GMO labeling regulations, initially mandating labeling when the GMO content in a food ingredient reached 1%, and later lowering this threshold to 0.9% in 2003 (Bruetschy, 2019). Countries such as Brazil, Australia, and New Zealand require labeling for products with GMO content exceeding 1% (Yao et al., 2025). In South Korea, the current permissible level for unintentional mixing is 3%, and it will be lowered to 0.9% by the end of 2026 (MFDS, 2025). In Japan, any ingredient that ranks among the top three on the ingredient list and exceeds 5% of the content are subject to mandatory labeling. In 2023, the standards for non-GMO labeling have been tightened, requiring that genetically modified components be non-detectable (Hino, 2002; Noguchi et al., 2016; Soga et al., 2022). In 2022, the United States issued the National Bioengineered Food Disclosure Standard, which mandates the labeling of bioengineered foods and sets the threshold at 5% (USDA, 2018; Buchman and Kovak, 2025). China currently employs a qualitative labeling approach and has proposed a quantitative threshold of 3% for single-crop GMO ingredients in products listed in the management catalog (MARA, 2023).

To support the implementation of labeling thresholds, quantitative real-time polymerase chain reaction (qPCR) is widely used for the absolute quantification of GMO content in samples (Milavec et al., 2014; Yao et al., 2024). Absolute quantification involves the simultaneous analysis of the test sample alongside a series of diluted standard samples with known contents. Based on the linear relationship between the Ct values of these standard samples and the initial template quantities, a standard curve is constructed. The content of the test sample can then be determined by calculating the initial template quantity from the measured Ct value using the standard curve or regression equation (Shin et al., 2023). However, this absolute quantification method requires the construction of a standard curve for each detection, making the procedure cumbersome, labor-intensive, costly, and demanding high analytical capabilities from the testing laboratory. Therefore, to support effective GMO labeling threshold management, there is an urgent need to develop more efficient, accurate, and cost-effective detection methods.

The comparative Ct method is similar to the calibration curve method, except that it uses mathematical formulas to achieve relative quantitative results (Schmittgen and Livak, 2008; Soga et al., 2022). In relative quantification, qPCR data are expressed relative to a reference gene (often a housekeeping gene). The ΔCt value represents the difference in Ct values between the target gene and the reference gene within the same sample, while the ΔΔCt value is the difference between the ΔCt values of two samples (e.g., a test sample and a calibrator or QC sample). In gene expression studies, it is often sufficient to know the difference in expression levels of a gene at different times or in different tissues or organs—for example, that the expression of a gene increases fivefold in one tissue compared to another. Livak and Schmittgen described the derivation, assumptions, and applications of the ΔΔCt method for analyzing gene expression data (Livak and Schmittgen, 2001). With this mathematical model, we enable quantification of GMO content in the samples. Specifically, this was achieved by measuring the relative difference in Ct values between two types of samples—the test sample and the QC sample—to quantify the content of GMO.

In GMO labeling threshold management, it is only necessary to determine whether the GMO content in a sample is above or below the labeling threshold; in most cases, precise quantification is not required. Based on the principles of the comparative Ct method, this study established a rapid ΔΔCt method for determining GMO content. By incorporating QC samples with known GMO content calibrated to the labeling threshold, the GMO content of the test sample is calculated. The ΔCt value is defined as the difference between the Ct values of the transgene and the reference gene within each sample, and the ΔΔCt value represents the difference between the ΔCt values of the test and QC samples. The GMO content is rapidly assessed based on the magnitude of the ΔΔCt value. In this study, following the criteria for key parameters of absolute quantification outlined in *Definition of Minimum Performance Requirements for Analytical Methods of GMO Testing* (ENGL, 2015), and in combination with the ΔΔCt method, we derived a cutoff value for ΔΔCt. This cutoff was subsequently validated through a multi-laboratory collaborative study, confirming that ΔΔCt = ± 0.4 constitutes the reliable threshold. Using this cutoff value, the GMO content in a sample can be rapidly determined: ΔΔCt < -0.4 indicates GMO content significantly above the labeling threshold; ΔΔCt > 0.4 indicates GMO content significantly below the labeling threshold; -0.4 ≤ ΔΔCt ≤ 0.4 indicates GMO content close to the labeling threshold, in which case standard curve-based absolute quantification should be performed if accurate measurement is required. The reliability and reproducibility of this cutoff were rigorously validated through an inter-laboratory collaborative study involving eight independent testing laboratories across two major GM crop matrices (soybean and maize), encompassing a wide range of GMO content levels. Our approach eliminates the need for standard curve construction, thereby significantly simplifying the workflow. Notably, while designed for rapid threshold-based screening, the method also enables a rough semi-quantitative estimation of GMO content, offering dual functionality well-suited for high-throughput regulatory testing. Collectively, these features provide a practical, cost-effective, and technically robust solution to support the enforcement of GMO labeling regulations, facilitating reliable compliance monitoring with minimal analytical complexity.

## 2 Materials and methods

### 2.1 Experimental materials

The experimental materials included three types of genetically modified (GM) crop samples. For GM soybean ZH6106, we prepared a standard sample (100% GMO content), a QC sample (1% GMO content), test samples with GMO levels of 5%, 2%, 1%, 0.5% and 0.1%, and a negative control sample of non-transgenic soybean Zhonghuang 10. For GM maize BFL4-2, the sample set contained a standard sample (100% GMO content), a QC sample (1% GMO content), test samples with GMO levels of 5%, 2%, 1%, 0.5% and 0.1%, and a negative control sample of non-transgenic maize Zheng 58. For GM maize BBL2-2, we used a standard sample (100% GMO content), a QC sample (3% GMO content), test samples with GMO levels of 5%, 3%, 1%, 0.5% and 0.1%, alongside a negative control sample of non-transgenic maize Zheng 58.

All samples were genomic DNA extracts prepared by our laboratory. Plant Genomic DNA Extraction Kit was purchased from TIANGEN Co. Ltd. (DP305, CHINA). Test samples were distributed as blind samples. The GMO contents of these samples were confirmed through inter-laboratory collaborative determination.

All primer and probe sequences, sourced from the Chinese national standards (**Supplementary Table 1**), were synthesized by Sangon Biotech (Shanghai) Co., Ltd. The qPCR reaction enzymes were provided by the collaborative validation laboratory. The qPCR instruments used were verified/calibrated in accordance with the requirements of the testing laboratory.

### 2.2 Experimental methods

#### 2.2.1 Sample content testing

Our organization coordinated eight GMO testing laboratories to perform quantitative detection on samples of three materials (GM soybean ZH6106, GM maize BFL4-2, and GM maize BBL2-2) with different GMO content levels. Each material included GMO samples at five content levels: 5%, 3% or 2%, 1%, 0.5%, and 0.1%. For each sample, three subsamples (independently collected from random locations within the same sample) were prepared for separate DNA extractions. Each DNA extract was then assayed in triplicate technical replicates (three parallel qPCR wells loaded with identical template from the same subsample). This experimental design was implemented to minimize variability arising from DNA extraction procedures and pipetting errors.

During testing, PCR amplification of both the transgene and the reference gene was conducted on the same PCR plate. Each laboratory used standard samples to generate a standard curve. In addition to the standard samples, each PCR plate included blank controls, negative controls, QC samples, and test samples. Standard curve data were collected based on the number of PCR plates used. The plate acceptance criteria were defined as follows: no amplification was observed in blank controls; for negative controls, the Ct value of the reference gene was ≤36 and the Ct value of the transgene was >36; key parameters of the standard curve (slope, coefficient of determination, amplification efficiency) met the specified requirements; and the measured values of QC samples fell within the allowable deviation range.

The primer-probe sets, PCR amplification system and program used in the experiment are provided in the **Supplementary Materials** (**Supplementary Tables 1**-**S3**).

#### 2.2.2 ΔΔCt method calculation

The Ct value data were collected according to the number of PCR plates used. The Ct values for both the QC samples and test samples on each PCR plate were statistically analyzed, encompassing the Ct values of the transgene and the reference gene.

Firstly, calculate the difference between the average Ct value of the transgene and the average Ct value of the reference gene for each sample; this value is the ΔCt (Equation 1). Then, calculate the difference between the average ΔCt value of the test sample and the average ΔCt value of the QC; this value is the ΔΔCt (Equation 2). Here, the average Ct value is the mean of three PCR replicates, while the average ΔCt value is the mean of measurements from three subsamples. The basic formulas for the ΔΔCt method are as follows:

(1) $$\Delta Ct=\bar{C}t_{GM}-\bar{C}t_{R}$$

(2) $$\Delta \Delta Ct=\Delta \bar{C}t_{S}-\Delta \bar{C}t_{QC}$$

Here: $\bar{C}t_{GM}$ is the average Ct value of the transgene, $\bar{C}t_{R}$ is the average Ct value of the reference gene, $\Delta \bar{C}t_{S}$ is the average ΔCt value of the test sample, $\Delta \bar{C}t_{QC}$ is the average ΔCt value of the QC sample.

A more intuitive representation of the formula is shown as follows:

(3) $$\Delta \Delta \text{Ct}={\left({\text{Ct}}_{\text{GM}}-{\text{Ct}}_{\text{R}}\right)}_{\text{test sample}}-{\left({\text{Ct}}_{\text{GM}}-{\text{Ct}}_{\text{R}}\right)}_{\text{QC sample}}$$

#### 2.2.3 Extended applications of the ΔΔCt method

The ΔΔCt method is used to calculate the change in GMO content based on the relative fold difference between the test sample and the QC sample. The GMO content can be calculated using the following formula:

(4) $${\text{CS}}_{=}{\left(1+\text{E}\right)}^{-\text{ΔΔCt}}\times {\text{C}}_{\text{QC}}$$

Here: C_S_ is the GMO content in the test sample, C_QC_ is the GMO content in the QC sample; E is the qPCR amplification efficiency, which should fall within the range of 0.9 to 1.1.

## 3 Results

### 3.1 Verification of sample content accuracy

The samples used in this study were laboratory-prepared rather than certified reference materials and were subsequently employed to verify the accuracy of the ΔΔCt cutoff value. Although internal verification of sample contents had been completed in the preliminary stage, eight collaborating laboratories were organized to confirm these contents via the standard curve quantification method, thereby enhancing the scientific rigor of this research and guaranteeing the accuracy of the ΔΔCt calculations. It should be noted that this confirmation procedure is not needed for practical implementation of the ΔΔCt method.

#### 3.1.1 Standard curve analysis and acceptance criteria

The quality of the standard curve directly affects the quantitative accuracy of GMO content. Analysis and verification of the standard curve to ensure its compliance with relevant requirements is a prerequisite for guaranteeing the accuracy and reliability of quantitative results of GMO content. This practice can effectively control systematic errors during the detection process and provide reliable data support for the subsequent determination of GMO content.

In accordance with *Definition of Minimum Performance Requirements for Analytical Methods of GMO Testing* (ENGL, 2015) and *Detection of Genetically Modified Organisms and Derived Products-technical Guidelines for the Development of Real-time Quantitative PCR methods* (MOA, 2015), the acceptance criteria for the standard curves of both the transgene and the reference gene are as follows: The slope (b) must fall within the range of -3.6 to -3.1; The coefficient of determination (R²) must have a minimum acceptable value of 0.98; The PCR amplification efficiency (E) must be within the range of 0.9 to 1.1. Only standard curves meeting all the above requirements can be used for subsequent analysis.

The statistical results of the standard curves are shown in **Supplementary Table 4**. All target and reference gene assays from eight collaborating laboratories exhibited amplification efficiencies ranging from 0.9 to 1.1 with consistent amplification kinetics, satisfying the aforementioned standard requirements. On this basis, the standard curve method was employed to quantify the GMO content in the samples.

Eight laboratories independently tested identical samples that were distributed to them. For each content level, three subsamples were analyzed, with each subsample subjected to at least three replicate PCR reactions. The Ct values for each content level were recorded, and the GMO content of the samples was calculated accordingly.

#### 3.1.2 Validation of data validity

In accordance with *Accuracy (trueness and precision) of measurement methods and results — Part 2: Basic method for the determination of repeatability and reproducibility of a standard measurement method* (SAC, 2004), Cochran’s test and Grubbs’ tests were employed to detect potential outliers within the test data from each laboratory. If the test statistic is less than or equal to the 5% critical value, the tested item is accepted as a correct value. If the test statistic is greater than the 5% critical value but less than or equal to the 1% critical value, the tested item is classified as a deviation value. This deviation value is retained and used in subsequent calculations. If the test statistic is greater than the 1% critical value, the tested item is identified as a statistical outlier. This outlier must be eliminated.

Measurement data from eight laboratories on 15 GMO samples were confirmed as valid after Cochran’s test and Grubbs’ test. The summarized results are presented in **Table 1**. The accuracy of the assigned values for sample content includes both trueness and precision. The results in **Table 1** show that for the 15 GMO samples (comprising 3 materials, each with 5 different content levels), the mean relative bias of the measurements from the eight laboratories fell within the ±25% range, meeting the trueness requirement. The relative repeatability standard deviation was less than 25%, and the relative reproducibility standard deviation was less than 35%, meeting the precision requirements. The purpose of the above data analysis is to ensure the accuracy, consistency, and reliability of the sample content.

**Table 1:** Inter-laboratory collaborative verification results for GMO content quantification.

Test Sample	GM soybean ZH6106					GM maize BFL4-2					GM maize BBL2-2				
Expected GMO content(%)	5.0	2.0	1.0	0.5	0.1	5.0	2.0	1.0	0.5	0.1	5.0	3.0	1.0	0.5	0.1
Number of data reporting laboratories	8	8	8	8	8	8	8	8	8	8	8	8	8	8	8
Number of subsamples	3	3	3	3	3	3	3	3	3	3	3	3	3	3	3
Number of outliers	0	0	0	0	0	0	0	0	0	0	0	0	0	0	0
Data analysis method	Cochran’s method and Grubbs’ method														
Measurement average value(%)	5.107	2.081	1.034	0.492	0.105	4.950	1.983	0.983	0.480	0.100	4.935	2.803	0.905	0.463	0.099
Relative repeatability standard deviation (RSD_r_)(%)	4.525	2.236	4.704	4.207	4.781	5.455	6.084	5.954	2.875	8.819	3.308	3.633	5.497	4.550	7.960
Relative reproducibility standard deviation (RSD_R_)(%)	6.016	8.280	8.765	6.950	7.655	9.317	9.878	9.474	9.458	9.315	6.695	7.254	9.742	10.583	9.493
Relative bias (biasR)(%)	2.140	4.050	3.400	-1.600	5.000	-1.008	-0.833	-1.667	-4.000	0.000	-1.308	-6.556	-9.542	-7.333	-1.083

A total of eight laboratories participated, with three subsamples per content level and at least three PCR replicates per subsample. Outlier detection was performed using Cochran’s and Grubbs’ tests at the 5% and 1% significance levels; no statistical outliers were identified. The acceptable ranges for trueness (|biasR| ≤ 25%) and precision (RSD_r_ ≤ 25%, RSD_R_ ≤ 35%) were based on ENGL (2015) and MOA (2015) guidelines.

### 3.2 Statistical analysis of ΔΔCt values

#### 3.2.1 Selection of reference gene and QC sample for the ΔΔCt method

The ΔΔCt method requires the selection of an appropriate reference gene to serve as an internal control. This reference gene should be a species-specific gene. Both the transgenic sequence and the reference gene are amplified simultaneously by qPCR, and the Ct values are recorded. Detection methods should prioritize those specified in standards or regulations. If no standard method is available, the detection methods for both the transgenic sequence and the reference gene should be properly validated to ensure that they have similar amplification efficiencies (Livak and Schmittgen, 2001). In this experiment, the *zSSIIb* gene was used as the reference gene for maize, and the *Lectin* gene for soybean. The detection methods for reference genes and GM events were developed in accordance with Chinese national standards (**Supplementary Table 1**); their specificity, limit of detection, amplification efficiency and other performance parameters were fully validated during standard formulation.

The QC sample is a positive control sample used for the rapid determination of GMO content. Certified reference materials should be prioritized for use, although validated laboratory-prepared samples may also be used. The measurement uncertainty of the standard value for the QC sample shall encompass the labeled threshold. In the absence of certified reference materials, the QC samples employed in this experiment were specimens that underwent inter-laboratory validation. The content test data for the QC samples are shown in **Supplementary Table 5**.

#### 3.2.2 Derivation of ΔΔCt cutoff values

**Figure 1:**
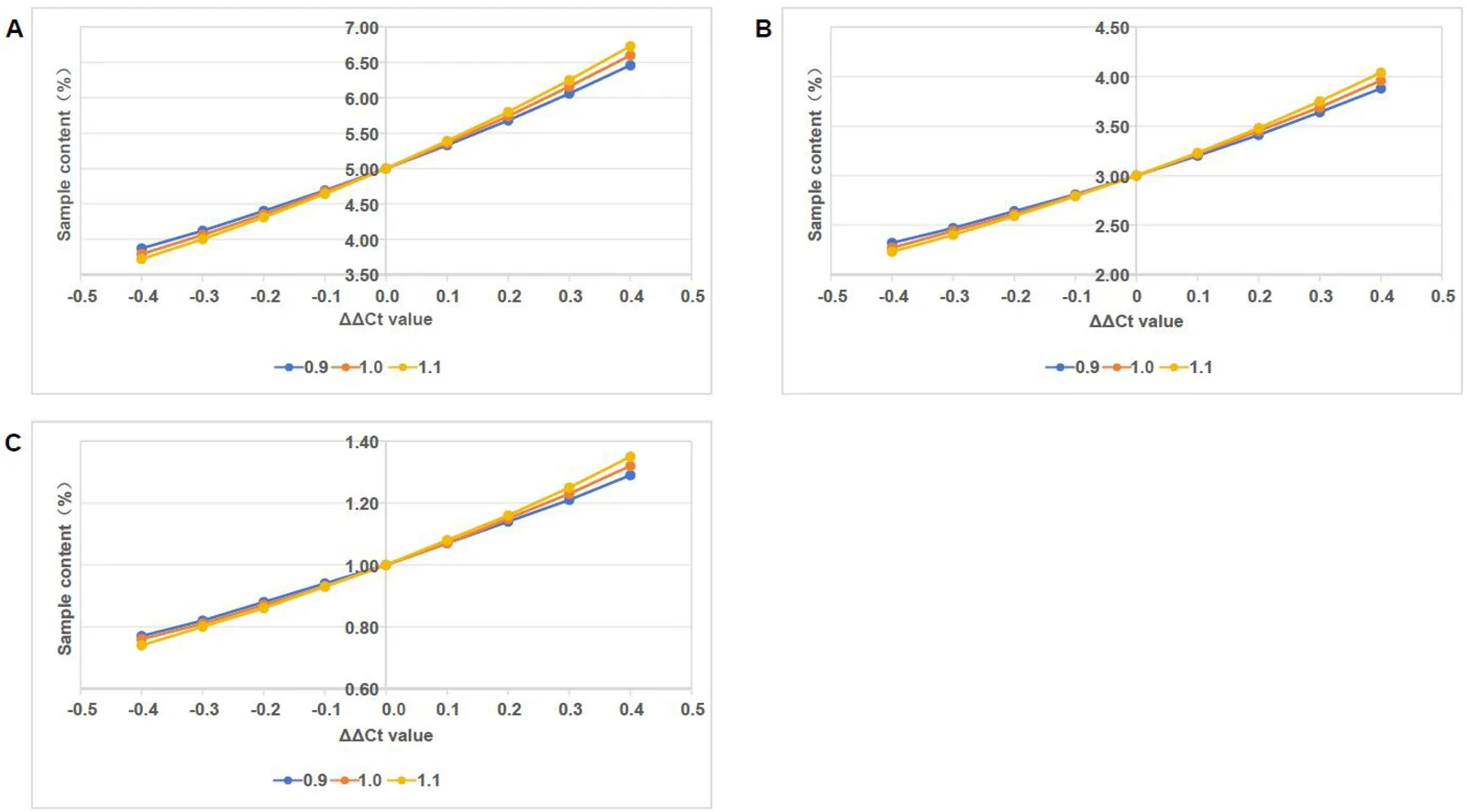
Relationship between ΔΔCt values and measured GMO content under different amplification efficiencies. The QC sample content was set at 5% **(A)**, 3% **(B)**, and 1% **(C)**, corresponding to commonly used labeling thresholds in different countries. Blue, orange, and yellow lines represent amplification efficiencies of 0.9, 1.0, and 1.1, respectively. As the absolute ΔΔCt value increases, the range of the measured GMO content broadens, particularly at higher amplification efficiencies.

According to Equation 4, the corresponding relationship between ΔΔCt values and sample content was calculated for samples with different amplification efficiencies and different content levels. The calculation results are presented in **Table 2**; **Figure 1**.

**Table 2:** Relationship between ΔΔCt values and measured GMO content under different amplification efficiencies.

GMO content		5%			3%			1%		
Amplification efficiency		0.9	1.0	1.1	0.9	1.0	1.1	0.9	1.0	1.1
ΔΔCt values	-0.4	3.87%	3.79%	**3.72%**	2.32%	2.27%	**2.23%**	0.77%	0.76%	**0.74%**
	-0.3	4.12%	4.06%	4.00%	2.47%	2.44%	2.40%	0.82%	0.81%	0.80%
	-0.2	4.40%	4.35%	4.31%	2.64%	2.61%	2.59%	0.88%	0.87%	0.86%
	-0.1	4.69%	4.67%	4.64%	2.81%	2.80%	2.79%	0.94%	0.93%	0.93%
	0	5.00%	5.00%	5.00%	3.00%	3.00%	3.00%	1.00%	1.00%	1.00%
	0.1	5.33%	5.36%	5.39%	3.20%	3.22%	3.23%	1.07%	1.07%	1.08%
	0.2	5.68%	5.74%	5.80%	3.41%	3.45%	3.48%	1.14%	1.15%	1.16%
	0.3	6.06%	6.16%	6.25%	3.64%	3.69%	3.75%	1.21%	1.23%	1.25%
	0.4	6.46%	6.60%	**6.73%**	3.88%	3.96%	**4.04%**	1.29%	1.32%	**1.35%**

The ΔΔCt values were calculated using Equation 4 with QC sample contents of 5%, 3%, and 1%, respectively. Bold values indicate the upper and lower boundaries defined by the selected cutoff of ±0.4. For example, at a labeling threshold of 1%, ΔΔCt values ranging from −0.4 to 0.4 correspond to a measured GMO content range of 0.74%–1.35%, which covers the required acceptance interval of 0.75%-1.25% according to ENGL (2015) and MOA (2015) guidelines.

According to *Definition of Minimum Performance Requirements for Analytical Methods of GMO Testing* (ENGL, 2015) and *Detection of Genetically Modified Organisms and Derived Products-technical Guidelines for the Development of Real-time Quantitative PCR methods* (MOA, 2015), the measured value of GMO content must not deviate from the standard value by more than 25%. This means: For a sample with a specified GMO content of 5%, the acceptable measurement range is 3.75% to 6.25%; For a specified content of 3%, the acceptable range is 2.25% to 3.75%; For a specified content of 1%, the acceptable range is 0.75% to 1.25%. Based on the data presented in **Table 2**; **Figure 1**, a larger absolute ΔΔCt value corresponds to higher amplification efficiency and a broader range of actual measured GMO content. When the ΔΔCt value is within the range of ±0.4 and the amplification efficiency is between 0.9 and 1.1: The measured range for a 5% sample is 3.72% to 6.73%; The measured range for a 3% sample is 2.23% to 4.04%; The measured range for a 1% sample is 0.74% to 1.35%. Based on these two points, we have established a ΔΔCt cutoff value of ±0.4. This ensures that the calculated sample content range, derived using this ΔΔCt cutoff value, covers the required measurement deviation range.

#### 3.2.3 Validation of ΔΔCt cutoff accuracy in practical testing

**Figure 2:**
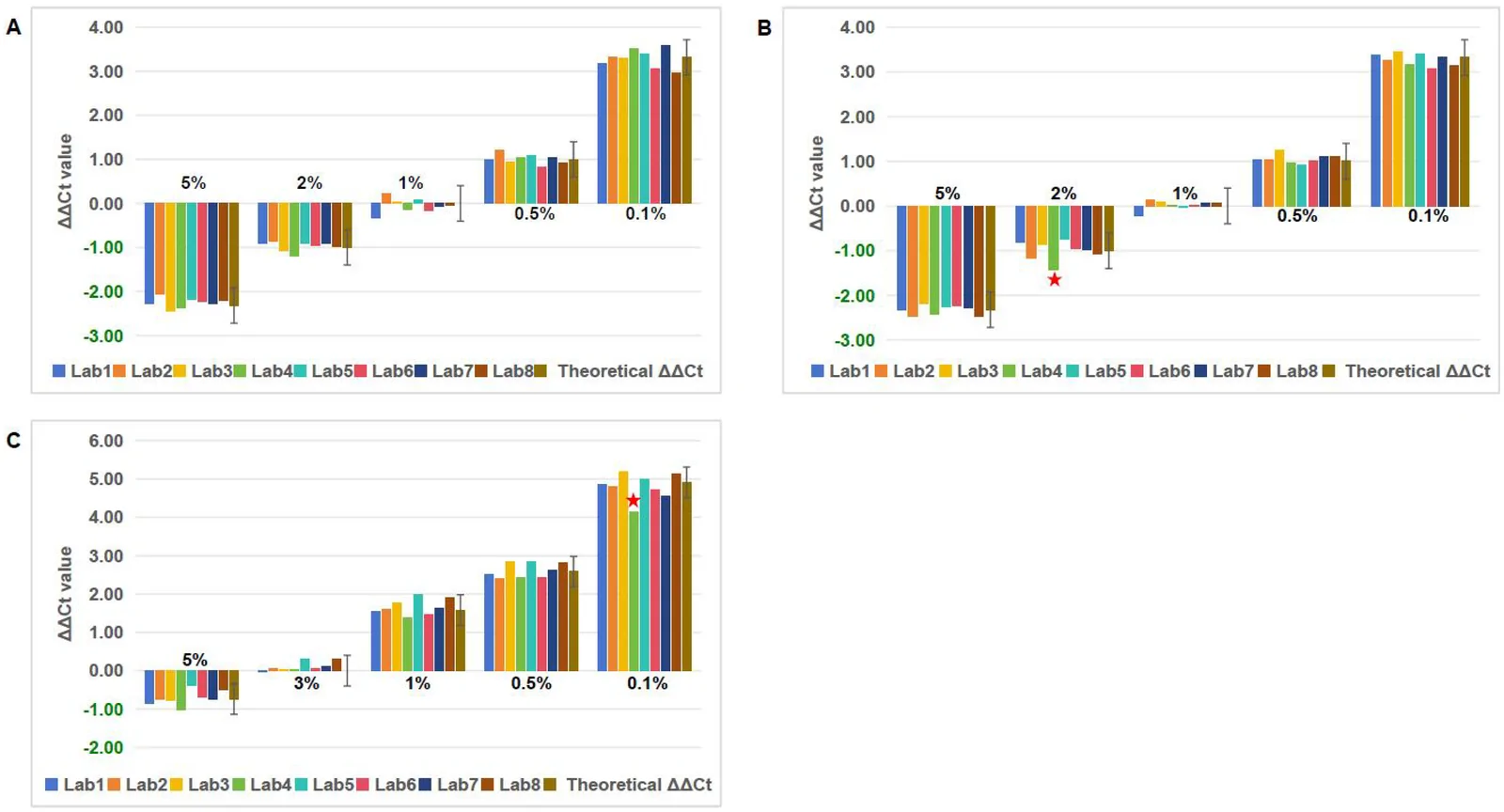
Inter-laboratory validation of ΔΔCt values for three GM events. **(A)** GM soybean ZH6106 (QC: 1% GMO); **(B)** GM maize BFL4-2 (QC: 1% GMO); **(C)** GM maize BBL2-2 (QC: 3% GMO). Each panel shows ΔΔCt values obtained from eight independent laboratories for test samples at different GMO content levels. Asterisks indicate data points that fell outside the theoretical ΔΔCt range (Lab4 for 2% BFL4–2 and Lab4 for 0.1% BBL2-2).

This study utilized only the measured Ct values from 15 GMO samples and the QC samples for data analysis to validate the accuracy of the derived ΔΔCt cutoff value. The ΔΔCt values for the test samples were calculated according to Equation 3. Statistical analysis of the raw data from the inter-laboratory collaborative validation is provided in the **Supplementary Materials** (**Supplementary Tables 6**-**S14**), while the summarized results of the ΔΔCt values are presented in **Table 3**; **Figure 2**.

**Table 3:** Summary of ΔΔCt values obtained from eight laboratories for 15 GMO test samples.

Lab	GM soybean ZH6106					GM maize BFL4-2					GM maize BBL2-2				
	5%	2%	1%	0.5%	0.1%	5%	2%	1%	0.5%	0.1%	5%	3%	1%	0.5%	0.1%
Lab1	-2.28	-0.91	-0.32	0.98	3.18	-2.33	-0.81	-0.22	1.03	3.37	-0.87	-0.02	1.53	2.50	4.86
Lab2	-2.07	-0.86	0.23	1.20	3.32	-2.47	-1.16	0.13	1.04	3.26	-0.74	0.05	1.59	2.40	4.79
Lab3	-2.45	-1.08	0.04	0.95	3.30	-2.19	-0.87	0.09	1.25	3.46	-0.78	0.02	1.77	2.84	5.19
Lab4	-2.38	-1.20	-0.15	1.05	3.52	-2.42	-1.44	0.01	0.97	3.17	-1.03	0.04	1.37	2.43	4.13
Lab5	-2.19	-0.90	0.08	1.09	3.39	-2.25	-0.74	-0.03	0.92	3.40	-0.38	0.30	1.98	2.84	5.00
Lab6	-2.22	-0.96	-0.16	0.83	3.06	-2.23	-0.95	0.01	1.01	3.06	-0.69	0.05	1.47	2.44	4.71
Lab7	-2.27	-0.91	-0.07	1.05	3.59	-2.27	-0.97	0.07	1.10	3.33	-0.74	0.11	1.64	2.62	4.56
Lab8	-2.20	-0.98	-0.05	0.91	2.95	-2.47	-1.08	0.06	1.10	3.14	-0.51	0.30	1.91	2.82	5.14
Mean ± SD	-2.26 ± 0.12	-0.97 ± 0.12	-0.05 ± 0.17	1.01 ± 0.11	3.29 ± 0.22	-2.33 ± 0.11	-1.00 ± 0.22	0.02 ± 0.11	1.05 ± 0.11	3.27 ± 0.14	-0.72 ± 0.20	0.11 ± 0.13	1.66 ± 0.20	2.61 ± 0.17	4.80 ± 0.33
Theoretical ΔΔCt	-2.32 ± 0.4	-1.00 ± 0.4	0.00 ± 0.4	1.00 ± 0.4	3.32 ± 0.4	-2.32 ± 0.4	-1.00 ± 0.4	0.00 ± 0.4	1.00 ± 0.4	3.32 ± 0.4	-0.74 ± 0.4	0.00 ± 0.4	1.58 ± 0.4	2.58 ± 0.4	4.91 ± 0.4

The QC sample content was 1% for GM soybean ZH6106 and GM maize BFL4-2, and 3% for GM maize BBL2-2. Data are presented as mean ΔΔCt values from three subsamples, each with three PCR replicates per laboratory. The theoretical ΔΔCt values were calculated based on amplification efficiencies ranging from 0.9 to 1.1. ΔΔCt values that fell outside the theoretical range are marked with asterisks in **Figure 2**.

The key focus for evaluating the accuracy of the ΔΔCt cutoff value is whether samples with GMO contents close to the labeling threshold (i.e., the content of the QC samples) can be correctly discriminated. As demonstrated in the tests of GM soybean ZH6106 with a QC content of 1%: test samples at 2% and 5% exhibited ΔΔCt < −0.4, indicating that their GMO contents were significantly higher than that of the QC sample; test samples at 0.1% and 0.5% showed ΔΔCt > 0.4, indicating that they were significantly lower than that of the QC sample; the 1% test sample had |ΔΔCt| ≤ 0.4, and its GMO content was judged to be comparable to that of the QC sample. These judgements were fully consistent with expectations. Identical results were obtained for GM maize BFL4-2 and BBL2-2. Therefore, the ΔΔCt cutoff value of 0.4 achieves high accuracy and meets the requirements for routine testing.

The theoretical ΔΔCt values for different GMO contents are presented in **Table 3**. These theoretical values were calculated using Equation 4. For Lab 4, the ΔΔCt values of the test samples of 2% GM maize BFL4-2 and 0.1% GM maize BBL2-2 deviated from the theoretical values, which was presumably attributable to operational errors in the laboratory. However, since these two outliers were not “close-to-threshold” values, they did not affect the determination of whether the GMO content of the test samples was above or below the labeling threshold using the ΔΔCt method.

### 3.3 Application of the ΔΔCt method

#### 3.3.1 Determination of GMO content

Based on the aforementioned ΔΔCt cutoff of ±0.4, the results generated by the ΔΔCt method can be rapidly interpreted as follows: If ΔΔCt < -0.4, it indicates that the GMO content in the sample is significantly higher than that of the QC sample;If ΔΔCt > 0.4, it indicates that the GMO content in the sample is significantly lower than that of the QC sample; If -0.4 ≤ ΔΔCt ≤ 0.4, it indicates that the GMO content in the sample is equivalent to that of the QC sample.

Here, we can select a QC sample with a content matching the labeling threshold. The ΔΔCt method can assist in rapidly determining whether the GMO content in the sample exceeds the labeling threshold.

#### 3.3.2 Calculation of GMO content

The GMO content can be calculated according to Equation 4. However, no standard curve is generated in the application of the ΔΔCt method, and the amplification efficiency E is unknown. For ease of application, we assume an ideal qPCR amplification condition with E = 1.0 to simplify the formula. Accordingly, the GMO content can be estimated approximately using Equation 5.

(5) $${\text{CS}}_{=}2^{-\text{ΔΔCt}}\times {\text{C}}_{\text{QC}}$$

here: ${\text{C}}_{S}$ is the GMO content in the test sample, ${\text{C}}_{\text{QC}}$ is the GMO content in the QC sample.

**Figure 3:**
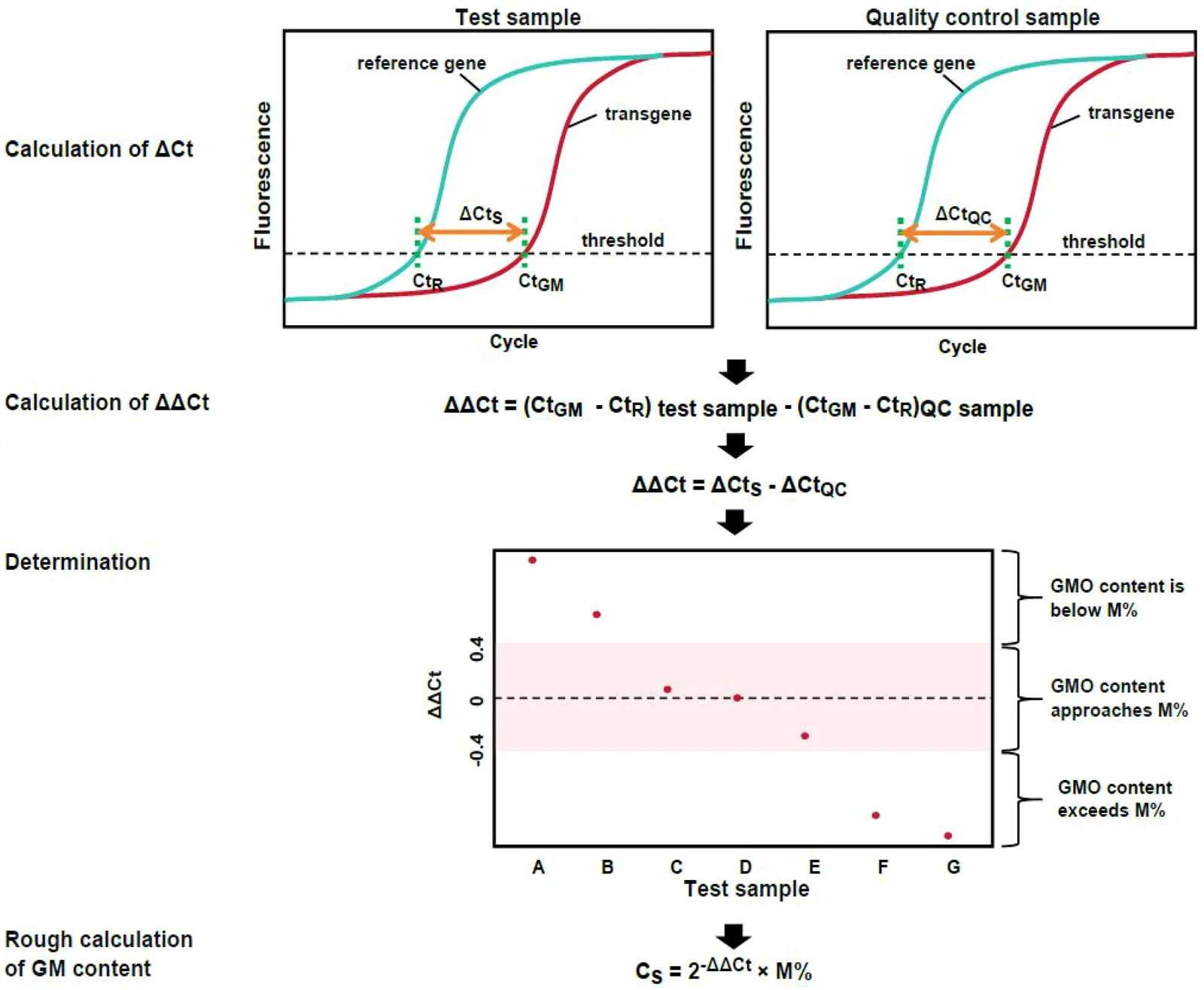
Workflow and decision criteria for GMO content assessment using the ΔΔCt method. The ΔCt values of the test sample (ΔCt_S_) and the QC sample (ΔCt_QC_) are determined by qPCR targeting the reference gene and the transgene. The ΔΔCt value is then calculated. The content of the QC sample can be set to be equivalent to the labeling threshold (e.g., 5% in the United States, 3% in China), denoted here as M%. The results are determined as follows: The GMO content of test samples A and B is significantly lower than M%, as these samples show ΔΔCt > 0.4. The GMO content of test samples F and G is significantly higher than M%, as these samples show ΔΔCt < -0.4. The GMO content of test samples C, D, and E is close to M%, as these samples show -0.4 ≤ ΔΔCt ≤ 0.4. Additionally, if necessary, the GMO content can be roughly calculated.

The two aforementioned applications of the ΔΔCt method can only determine the approximate range of GMO content. For accurate quantification, absolute quantification methods involving the construction of a standard curve can be employed to obtain precise GMO content. **Figure 3** shows the workflow for GMO content detection using the ΔΔCt method.

## 4 Discussion

Conventional GMO quantification relies on absolute qPCR via standard curves. These methods utilize standard curves to provide data and convert it into precise copy numbers, which adds the workload of generating standard curves to the process (Schmittgen and Livak, 2008; Chen et al., 2005). However, in the management of GMO labeling thresholds, it is often unnecessary to show data in terms of absolute GMO content; instead, it only requires determining whether the GMO content in the sample is above or below the labeling threshold. The ΔΔCt-based qPCR method is a relative quantification approach that enables rapid calculation of GMO content by using the QC sample with known GMO content (Noguchi et al., 2016).

The relative quantification method incorporates the PCR efficiencies of both the target gene and the reference gene into the equation, thereby accounting for differences in their amplification efficiencies during calculation (Liu and Saint, 2002; Rutledge, 2004). The ΔΔCt method, also known as the comparative CT method, employs a mathematical formula to derive relative quantification results by calculating the change in GMO content based on the relative fold difference between the test sample and the calibrator. Under ideal qPCR amplification conditions, the amplification efficiency is 1.0. The ΔΔCt method is a simplified approach that assumes the amplification efficiencies of both the target gene and the reference gene are identical and equal to 1.0. This method is convenient to use as it eliminates the need to calculate amplification efficiency through serial dilutions. However, in this study, we did not rely entirely on this simplifying assumption. For calculations using Equation 4, we corrected deviations from the ideal amplification efficiency of 1.0 and accounted for associated analytical errors. Specifically, the amplification efficiency should fall within the range of 0.9 to 1.1, and the amplification kinetics of the target and reference genes should be approximately equal. Additionally, the use of the ΔΔCt method requires calculation based on Ct data. It should be emphasized that statistical analysis should not be performed directly on the raw Ct data. Typically, each sample requires both PCR replicates and sample replicates (subsamples). The analysis should begin by averaging these PCR replicates, followed by calculating the mean value for subsamples. This two-tier averaging strategy effectively minimizes errors arising from experimental operations. This optimized calculation framework laid a solid foundation for the subsequent validation of the ΔΔCt cutoff criterion via inter-laboratory collaborative tests.

The accuracy of GMO content measurement results is generally evaluated based on two aspects: trueness and precision. Trueness refers to the degree of agreement between the average of a large number of test results and the true value or an accepted reference value, while precision refers to the degree of agreement among individual test results. According to *Definition of Minimum Performance Requirements for Analytical Methods of GMO Testing* (ENGL, 2015) and *Detection of Genetically Modified Organisms and Derived Products-technical Guidelines for the Development of Real-time Quantitative PCR methods* (MOA, 2015), the relative bias of measured values within a laboratory must not exceed 25% of the true value. Since the actual measurement results were all less than 10% (**Table 1**), setting the ΔΔCt cutoff value at ±0.4 can fully cover the deviation range of the measurement results. In this study, we validated the accuracy of the ΔΔCt cutoff value using actual detection data. Using this cutoff value allows accurate classification of three sample groups (above, below, or close to the labeling threshold) and satisfies routine testing demands. Owing to the simple experimental sample design, this method is well suited for large-scale screening of GMO samples. We further compared this cost-effective routine qPCR-based screening strategy with another mainstream standard-curve-free quantitative tool, digital PCR, to clarify their respective application niches.

In recent years, digital PCR (dPCR) has emerged as a novel molecular platform enabling absolute quantification without standard curves, and it has seen expanding applications in GMO detection (Demeke and Dobnik, 2018; Bogožalec Košir et al., 2019). Although our ΔΔCt method also avoids standard curve preparation, the two approaches differ greatly in working principles and applicable scenarios. Digital PCR relies on limiting dilution and Poisson distribution statistics to deliver high quantification precision and strong resistance to PCR inhibitors, rendering it ideal for low-abundance transgenic targets and certified reference material characterization (Morisset et al., 2013; Cleveland et al., 2024). Nevertheless, dPCR necessitates dedicated instruments, expensive consumables and prolonged testing durations, restricting its routine deployment for high-throughput screening. By contrast, the ΔΔCt strategy developed herein runs on conventional qPCR instruments widely accessible in testing facilities, incurs no extra hardware expenses and allows rapid analysis with trivial operational adjustments. While dPCR remains indispensable for precise quantification and method validation, our ΔΔCt assay is tailored exclusively for rapid threshold compliance screening, serving as a complementary rather than competing technique. The two tools fulfill separate analytical demands: dPCR for accurate absolute quantification and reference value calibration, and the ΔΔCt method for low-cost, large-scale screening to facilitate GMO labeling regulation implementation. Although the ΔΔCt method shows prominent advantages in batch threshold screening, its semi-quantitative estimation function has clear applicable constraints that require standardized laboratory operation.

In addition to enabling rapid determination of whether the GMO content in samples exceeds or falls below the labeling threshold, this study further extends the application of the ΔΔCt method to estimate GMO content in test samples. When employing the ΔΔCt method for rapid quantification of GMO content, the absolute amplification efficiency (E) cannot be determined for the current experiment, as no standard curve is constructed. However, the established and validated assays for both the target and reference genes yield amplification efficiencies ranging from 0.9 to 1.1. To estimate GMO content, we may assume an ideal qPCR amplification efficiency (E = 1.0). Using Equation 5, the GMO content in samples can be calculated from the ΔΔCt values of the test samples and QC samples. Nevertheless, the calculation of GMO content using this formula is applicable only under conditions where ΔΔCt < −0.4 or ΔΔCt > 0.4, corresponding to scenarios in which the GMO content is significantly higher or lower than that of the QC samples. Under such conditions, the calculated GMO content provides only an approximate estimate rather than an accurate quantitative determination. Notably, in cases where the observed ΔΔCt values deviate from the theoretical expectations, the calculation results are subject to considerable bias. Therefore, if Equation 5 is to be employed for GMO content quantification, testing laboratories should standardize experimental operations and implement appropriate quality control procedures to minimize the occurrence of large deviations. This semi-quantitative strategy, which eliminates the need for standard curve construction, greatly simplifies experimental procedures, reduces detection costs, provides robust technical support for threshold-based labeling management, and ensures the effective implementation of GMO regulatory systems.

It should be acknowledged that the present study focused on method validation using laboratory-prepared reference samples with well-characterized GMO contents, rather than commercial food products. This approach was intentionally chosen to establish the analytical performance of the ΔΔCt method under controlled conditions, where true GMO contents are known. Such a validation framework is a prerequisite for subsequent application to real food matrices. The successful inter-laboratory validation using 15 blind samples across three GM events lays a robust foundation for future studies targeting commercial food matrices. Nonetheless, we recognize that validation using commercial samples would further strengthen the practical applicability of this method, and this represents a logical next step in our ongoing work.

## 5 Conclusion

The enforcement of GMO labeling threshold regulations requires the development of rapid and cost-effective quantitative techniques. Conventional quantitative approaches, which rely on constructing standard curves for each assay, are often laborious and costly. This study presents a rapid quantification strategy based on parallel testing of target samples alongside QC samples corresponding to the labeling threshold.

By calculating the ΔΔCt value between samples and controls and applying a ±0.4 ΔΔCt cutoff, this method enables efficient assessment of GMO content: ΔΔCt < -0.4 indicates GMO content significantly above the labeling threshold; ΔΔCt > 0.4 indicates GMO content significantly below the labeling threshold; -0.4 ≤ ΔΔCt ≤ 0.4 indicates GMO content close to the labeling threshold. If needed, absolute quantification via a standard curve can be performed for precise determination. The accuracy and reproducibility of this method have been verified through multi-laboratory collaborative studies. In practice, especially for high-throughput sample testing, this rapid strategy can greatly improve efficiency and reduce costs, offering strong technical support for labeling threshold management.

## Data Availability

The original contributions presented in the study are included in the article/**Supplementary Material**. Further inquiries can be directed to the corresponding author.
