# Supplementary material for: A standard-curve-free ΔΔCt method for rapid GMO compliance screening relative to labeling thresholds
Source: Front Plant Sci. 2026 Aug 27;17:1918379. doi: 10.3389/fpls.2026.1918379 (PMC13558396; doi:10.3389/fpls.2026.1918379)
Supplement: Supplementary file 1 [file Table1.docx]

Supplementary Materials

**TABLE S1** Prime and probe information of GM events and reference genes.

| Gene/event | Primer/probe | Sequence (5′-3′) | Amplicon size (bp) | Source |
| --- | --- | --- | --- | --- |
| *Lectin* | lec-1215F  lec-1332R  lec-1269P | GCCCTCTACTCCACCCCCA  GCCCATCTGCAAGCCTTTTT  FAM-AGCTTCGCCGCTTCCTTCAACTTCAC-BHQ1 | 118 | MARA Announcement No. 2031-8-2013 |
| *zSSIIb* | zSSIIb-3F  zSSIIb-4R  zSSIIb-P | CGGTGGATGCTAAGGCTGATG  AAAGGGCCAGGTTCATTATCCTC  FAM-TAAGGAGCACTCGCCGCCGCATCTG-BHQ1 | 88 | MARA Announcement No. 1861-3-2012 |
| ZH6106 | ZH6106-qF  ZH6106-qR  ZH6106-qP | TCTAAGCGTCAATTTGTTTACATCA  AAAGGTGTGAAATGTGAACGC  FAM-TTACCTAAAACATCTCAGCACATCGC-BHQ1 | 96 | MARA Announcement No. 864-16-2024 |
| BFL4-2 | BFL4-2-QF  BFL4-2-QR  BFL4-2-QP | CGTCCGCAATGTGTTATT  GCAACTACTTTGGATGAGG  FAM-CTAAGCGTCAATTTGTTTACACCAC-BHQ1 | 98 | MARA Announcement No. 864-5-2024 |
| BBL2-2 | BBL2-2-QF  BBL2-2-QR  BBL2-2-QP | GTGATCTTGTTCGTTGTATTGG  TTCGGCGTTAATTCAGTACAT  FAM-TCCCTGAGAGTCAACTGCCCC-BHQ1 | 119 | MARA Announcement No. 1012-3-2026 |

**Footnote:** The *Lectin* gene is the reference gene for soybeans, and the *zSSIIb* gene is the reference gene for maize. All primer and probe sequences are from the Chinese national standards, with the standard numbers listed.

**TABLE S2** Real-time PCR amplification system

| Reagent | Final Concentration | Volume |
| --- | --- | --- |
| ddH_2_O |  | 8.0 *µ*L |
| PCR Buffer (2×) | 1× | 12.5 *µ*L |
| Forward Primer (10 µmol/L) | 0.4 *µ*mol/L | 1.0 *µ*L |
| Reverse Primer (10 µmol/L) | 0.4 *µ*mol/L | 1.0 *µ*L |
| Probe (10 µmol/L) | 0.2 *µ*mol/L | 0.5 *µ*L |
| DNA Template |  | 2.0 *µ*L |
| Total Volume |  | 25.0 *µ*L |

**TABLE S3 Real-time PCR** amplification program

| Step | Procedure | Cycles |
| --- | --- | --- |
| Stage 1 | 95°C Predenaturation for 5 min | 1 |
| Stage 2 | 95°C Denaturation for 15 s, 60°C Annealing and Extension for 60 s | ≥40 |

**TABLE S4** Statistical results of the standard curves **from Inter-laboratory Collaborative Validation**

| Lab | *Lectin* | | | ZH6106 | | | *zSSIIb* | | | BFL4-2 | | | *zSSIIb* | | | BBL2-2 | | |
| --- | --- | --- | --- | --- | --- | --- | --- | --- | --- | --- | --- | --- | --- | --- | --- | --- | --- | --- |
|  | b | R² | E  （%） | b | R² | E  （%） | b | R² | E  （%） | b | R² | E  （%） | b | R² | E  （%） | b | R² | E  （%） |
| Lab1 | -3.415 | 0.998 | 96.453 | -3.368 | 0.995 | 98.140 | -3.244 | 0.996 | 103.373 | -3.379 | 0.998 | 97.700 | -3.260 | 0.998 | 102.867 | -3.323 | 0.998 | 99.967 |
| Lab2 | -3.367 | 0.999 | 98.140 | -3.163 | 0.991 | 106.983 | -3.289 | 0.999 | 101.407 | -3.355 | 0.998 | 98.637 | -3.326 | 0.996 | 99.833 | -3.278 | 0.995 | 101.969 |
| Lab3 | -3.366 | 0.999 | 98.200 | -3.335 | 0.998 | 99.467 | -3.480 | 0.997 | 93.833 | -3.419 | 0.998 | 96.167 | -3.467 | 0.998 | 94.300 | -3.451 | 0.996 | 94.900 |
| Lab4 | -3.473 | 0.999 | 94.069 | -3.393 | 0.998 | 97.108 | -3.283 | 0.988 | 101.906 | -3.437 | 0.997 | 95.516 | -3.166 | 0.992 | 106.982 | -3.208 | 0.996 | 105.000 |
| Lab5 | -3.312 | 0.999 | 100.407 | -3.309 | 0.999 | 100.587 | -3.395 | 0.996 | 97.151 | -3.364 | 0.998 | 98.294 | -3.330 | 0.998 | 99.685 | -3.326 | 0.998 | 99.822 |
| Lab6 | -3.163 | 0.996 | 107.100 | -3.145 | 0.989 | 107.933 | -3.133 | 0.997 | 108.533 | -3.173 | 0.997 | 106.633 | -3.160 | 0.999 | 107.200 | -3.187 | 0.999 | 105.967 |
| Lab7 | -3.445 | 0.999 | 95.067 | -3.417 | 0.999 | 96.200 | -3.378 | 0.990 | 97.733 | -3.420 | 0.994 | 96.133 | -3.349 | 0.996 | 98.933 | -3.371 | 0.994 | 98.133 |
| Lab8 | -3.256 | 0.999 | 102.900 | -3.163 | 0.998 | 107.133 | -3.302 | 0.994 | 101.139 | -3.319 | 0.996 | 100.353 | -3.364 | 0.998 | 98.382 | -3.455 | 0.998 | 94.754 |

**TABLE S5** GMO content test data for quality control samples

| Sample | Subsample | Measured value of quality control samples (%) | | | | | | | | mean | RSD_R_（%） | Bias_R_（%） |
| --- | --- | --- | --- | --- | --- | --- | --- | --- | --- | --- | --- | --- |
|  |  | Lab1 | Lab2 | Lab3 | Lab4 | Lab5 | Lab6 | Lab7 | Lab8 |  |  |  |
| ZH6106 | 1 | 1.20 | 1.09 | 0.98 | 1.04 | 1.06 | 0.98 | 1.04 | 1.05 | 1.039 | 9.054 | 3.917 |
|  | 2 | 1.09 | 1.19 | 0.99 | 1.09 | 0.99 | 0.93 | 0.97 | 0.95 |  |  |  |
|  | 3 | 0.86 | 1.19 | 1.04 | 1.24 | 0.98 | 0.96 | 1.06 | 0.97 |  |  |  |
| BFL4-2 | 1 | 0.97 | 0.90 | 1.21 | 0.83 | 1.09 | 0.99 | 1.12 | 0.85 | 0.971 | 12.270 | -2.917 |
|  | 2 | 0.84 | 0.97 | 1.19 | 1.04 | 0.84 | 0.98 | 1.08 | 0.93 |  |  |  |
|  | 3 | 0.90 | 0.98 | 1.12 | 0.78 | 0.85 | 1.01 | 0.97 | 0.86 |  |  |  |
| BBL2-2 | 1 | 2.73 | 2.98 | 2.96 | 2.50 | 3.66 | 3.04 | 3.08 | 3.08 | 2.910 | 10.763 | -2.986 |
|  | 2 | 2.60 | 2.79 | 2.94 | 2.30 | 3.36 | 2.99 | 3.04 | 3.00 |  |  |  |
|  | 3 | 2.81 | 2.91 | 2.88 | 2.25 | 3.21 | 3.18 | 2.62 | 2.94 |  |  |  |

**TABLE S6** **Calculation of ΔΔCt Values from Inter-laboratory Collaborative Validation Data (GM Soybean ZH6106 at 5% and 2% Content)**

| Lab | Sample | | Reference gene Ct | Event Ct | ΔCt | ΔΔCt | Average ΔΔCt | Theoretical ΔΔCt |
| --- | --- | --- | --- | --- | --- | --- | --- | --- |
| Lab1 | QC（1%） | / | 23.41 | 30.61 | 7.20 | / | / | / |
|  | 5% | 1 | 23.66 | 28.63 | 4.98 | -2.22 | -2.28 | -2.32±0.4 |
|  |  | 2 | 23.66 | 28.66 | 5.00 | -2.20 |  |  |
|  |  | 3 | 23.78 | 28.57 | 4.79 | -2.41 |  |  |
|  | 2% | 1 | 23.35 | 29.63 | 6.28 | -0.92 | -0.91 | -1.00±0.4 |
|  |  | 2 | 23.36 | 29.70 | 6.34 | -0.86 |  |  |
|  |  | 3 | 23.35 | 29.60 | 6.25 | -0.95 |  |  |
| Lab2 | QC（1%） | / | 25.53 | 32.82 | 7.29 | / | / | / |
|  | 5% | 1 | 25.40 | 30.60 | 5.21 | -2.08 | -2.07 | -2.32±0.4 |
|  |  | 2 | 25.43 | 30.63 | 5.20 | -2.09 |  |  |
|  |  | 3 | 25.31 | 30.58 | 5.27 | -2.02 |  |  |
|  | 2% | 1 | 25.29 | 31.68 | 6.39 | -0.90 | -0.86 | -1.00±0.4 |
|  |  | 2 | 25.31 | 31.79 | 6.48 | -0.81 |  |  |
|  |  | 3 | 25.30 | 31.73 | 6.43 | -0.86 |  |  |
| Lab3 | QC（1%） | / | 24.31 | 33.02 | 8.72 | / | / | / |
|  | 5% | 1 | 24.13 | 30.36 | 6.23 | -2.49 | -2.45 | -2.32±0.4 |
|  |  | 2 | 24.18 | 30.46 | 6.28 | -2.44 |  |  |
|  |  | 3 | 24.23 | 30.51 | 6.28 | -2.43 |  |  |
|  | 2% | 1 | 24.14 | 31.82 | 7.67 | -1.04 | -1.08 | -1.00±0.4 |
|  |  | 2 | 24.12 | 31.72 | 7.60 | -1.12 |  |  |
|  |  | 3 | 24.16 | 31.79 | 7.63 | -1.09 |  |  |
| Lab4 | QC（1%） | / | 23.97 | 30.60 | 6.63 | / | / | / |
|  | 5% | 1 | 24.12 | 28.45 | 4.33 | -2.30 | -2.38 | -2.32±0.4 |
|  |  | 2 | 24.22 | 28.53 | 4.31 | -2.32 |  |  |
|  |  | 3 | 24.35 | 28.48 | 4.13 | -2.50 |  |  |
|  | 2% | 1 | 24.42 | 29.86 | 5.44 | -1.19 | -1.20 | -1.00±0.4 |
|  |  | 2 | 24.42 | 29.84 | 5.41 | -1.22 |  |  |
|  |  | 3 | 24.45 | 29.88 | 5.44 | -1.19 |  |  |
| Lab5 | QC（1%） | / | 22.79 | 28.74 | 5.94 | / | / | / |
|  | 5% | 1 | 22.92 | 26.76 | 3.84 | -2.10 | -2.19 | -2.32±0.4 |
|  |  | 2 | 22.94 | 26.64 | 3.70 | -2.24 |  |  |
|  |  | 3 | 22.99 | 26.70 | 3.71 | -2.23 |  |  |
|  | 2% | 1 | 22.94 | 27.95 | 5.01 | -0.93 | -0.90 | -1.00±0.4 |
|  |  | 2 | 22.87 | 27.97 | 5.10 | -0.84 |  |  |
|  |  | 3 | 22.86 | 27.88 | 5.02 | -0.92 |  |  |
| Lab6 | QC（1%） | / | 24.52 | 31.56 | 7.04 | / | / | / |
|  | 5% | 1 | 24.61 | 29.40 | 4.79 | -2.25 | -2.22 | -2.32±0.4 |
|  |  | 2 | 24.85 | 29.71 | 4.86 | -2.18 |  |  |
|  |  | 3 | 24.89 | 29.71 | 4.83 | -2.22 |  |  |
|  | 2% | 1 | 24.66 | 30.74 | 6.08 | -0.96 | -0.96 | -1.00±0.4 |
|  |  | 2 | 24.84 | 30.94 | 6.10 | -0.94 |  |  |
|  |  | 3 | 24.87 | 30.93 | 6.06 | -0.98 |  |  |
| Lab7 | QC（1%） | / | 21.66 | 28.39 | 6.73 | / | / | / |
|  | 5% | 1 | 21.52 | 25.96 | 4.45 | -2.29 | -2.27 | -2.32±0.4 |
|  |  | 2 | 21.48 | 25.93 | 4.45 | -2.29 |  |  |
|  |  | 3 | 21.53 | 26.04 | 4.51 | -2.22 |  |  |
|  | 2% | 1 | 21.54 | 27.29 | 5.75 | -0.98 | -0.91 | -1.00±0.4 |
|  |  | 2 | 21.50 | 27.37 | 5.87 | -0.87 |  |  |
|  |  | 3 | 21.49 | 27.34 | 5.85 | -0.89 |  |  |
| Lab8 | QC（1%） | / | 24.56 | 31.20 | 6.64 | / | / | / |
|  | 5% | 1 | 24.55 | 28.97 | 4.42 | -2.22 | -2.20 | -2.32±0.4 |
|  |  | 2 | 24.64 | 29.08 | 4.45 | -2.19 |  |  |
|  |  | 3 | 24.58 | 29.05 | 4.47 | -2.17 |  |  |
|  | 2% | 1 | 24.68 | 30.32 | 5.64 | -1.00 | -0.98 | -1.00±0.4 |
|  |  | 2 | 24.66 | 30.35 | 5.68 | -0.96 |  |  |
|  |  | 3 | 24.64 | 30.31 | 5.67 | -0.97 |  |  |

**Footnote:** For each content level, three subsamples were analyzed. Ct values of the reference gene and the transgene are the means of three PCR replicates. The same applies throughout.

**TABLE S7** **Calculation of ΔΔCt Values from Inter-laboratory Collaborative Validation Data (GM Soybean ZH6106 at 1% and 0.5% Content)**

| Lab | Sample | | Reference gene Ct | Event Ct | ΔCt | ΔΔCt | Average ΔΔCt | Theoretical ΔΔCt |
| --- | --- | --- | --- | --- | --- | --- | --- | --- |
| Lab1 | QC（1%） | / | 23.60 | 31.06 | 7.47 | / | / | / |
|  | 1% | 1 | 23.51 | 30.54 | 7.03 | -0.44 | -0.32 | 0.00±0.4 |
|  |  | 2 | 23.39 | 30.68 | 7.29 | -0.17 |  |  |
|  |  | 3 | 23.42 | 30.54 | 7.12 | -0.35 |  |  |
|  | 0.5% | 1 | 23.39 | 31.75 | 8.37 | 0.90 | 0.98 | 1.00±0.4 |
|  |  | 2 | 23.37 | 31.93 | 8.56 | 1.09 |  |  |
|  |  | 3 | 23.40 | 31.83 | 8.43 | 0.96 |  |  |
| Lab2 | QC（1%） | / | 25.07 | 32.53 | 7.47 | / | / | / |
|  | 1% | 1 | 24.98 | 32.64 | 7.66 | 0.20 | 0.23 | 0.00±0.4 |
|  |  | 2 | 24.98 | 32.74 | 7.76 | 0.29 |  |  |
|  |  | 3 | 24.99 | 32.67 | 7.67 | 0.21 |  |  |
|  | 0.5% | 1 | 24.98 | 33.65 | 8.67 | 1.20 | 1.20 | 1.00±0.4 |
|  |  | 2 | 25.09 | 33.75 | 8.66 | 1.20 |  |  |
|  |  | 3 | 25.01 | 33.67 | 8.66 | 1.19 |  |  |
| Lab3 | QC（1%） | / | 24.84 | 32.95 | 8.11 | / | / | / |
|  | 1% | 1 | 24.68 | 32.75 | 8.07 | -0.03 | 0.04 | 0.00±0.4 |
|  |  | 2 | 24.65 | 32.77 | 8.13 | 0.02 |  |  |
|  |  | 3 | 24.76 | 33.00 | 8.24 | 0.13 |  |  |
|  | 0.5% | 1 | 24.81 | 33.81 | 9.00 | 0.89 | 0.95 | 1.00±0.4 |
|  |  | 2 | 24.91 | 34.00 | 9.09 | 0.98 |  |  |
|  |  | 3 | 24.91 | 33.98 | 9.07 | 0.96 |  |  |
| Lab4 | QC（1%） | / | 24.45 | 30.72 | 6.28 | / | / | / |
|  | 1% | 1 | 24.48 | 30.67 | 6.19 | -0.09 | -0.15 | 0.00±0.4 |
|  |  | 2 | 24.40 | 30.53 | 6.13 | -0.14 |  |  |
|  |  | 3 | 24.61 | 30.67 | 6.06 | -0.21 |  |  |
|  | 0.5% | 1 | 24.69 | 32.02 | 7.33 | 1.06 | 1.05 | 1.00±0.4 |
|  |  | 2 | 24.62 | 31.84 | 7.22 | 0.95 |  |  |
|  |  | 3 | 24.63 | 32.06 | 7.42 | 1.15 |  |  |
| Lab5 | QC（1%） | / | 23.34 | 29.44 | 6.11 | / | / | / |
|  | 1% | 1 | 23.31 | 29.54 | 6.23 | 0.13 | 0.08 | 0.00±0.4 |
|  |  | 2 | 23.36 | 29.44 | 6.08 | -0.03 |  |  |
|  |  | 3 | 23.38 | 29.65 | 6.26 | 0.16 |  |  |
|  | 0.5% | 1 | 23.56 | 30.64 | 7.07 | 0.97 | 1.09 | 1.00±0.4 |
|  |  | 2 | 23.58 | 30.79 | 7.21 | 1.11 |  |  |
|  |  | 3 | 23.53 | 30.84 | 7.31 | 1.20 |  |  |
| Lab6 | QC（1%） | / | 24.49 | 31.56 | 7.07 | / | / | / |
|  | 1% | 1 | 24.67 | 31.56 | 6.89 | -0.18 | -0.16 | 0.00±0.4 |
|  |  | 2 | 24.60 | 31.53 | 6.93 | -0.14 |  |  |
|  |  | 3 | 24.63 | 31.54 | 6.91 | -0.16 |  |  |
|  | 0.5% | 1 | 24.98 | 32.85 | 7.87 | 0.80 | 0.83 | 1.00±0.4 |
|  |  | 2 | 24.77 | 32.62 | 7.85 | 0.78 |  |  |
|  |  | 3 | 24.77 | 32.73 | 7.96 | 0.89 |  |  |
| Lab7 | QC（1%） | / | 21.61 | 28.43 | 6.82 | / | / | / |
|  | 1% | 1 | 21.55 | 28.30 | 6.74 | -0.08 | -0.07 | 0.00±0.4 |
|  |  | 2 | 21.56 | 28.27 | 6.71 | -0.11 |  |  |
|  |  | 3 | 21.55 | 28.33 | 6.78 | -0.04 |  |  |
|  | 0.5% | 1 | 21.55 | 29.42 | 7.87 | 1.05 | 1.05 | 1.00±0.4 |
|  |  | 2 | 21.57 | 29.42 | 7.85 | 1.03 |  |  |
|  |  | 3 | 21.62 | 29.50 | 7.89 | 1.07 |  |  |
| Lab8 | QC（1%） | / | 24.85 | 31.25 | 6.40 | / | / | / |
|  | 1% | 1 | 24.78 | 31.08 | 6.30 | -0.10 | -0.05 | 0.00±0.4 |
|  |  | 2 | 24.82 | 31.10 | 6.28 | -0.12 |  |  |
|  |  | 3 | 24.57 | 31.04 | 6.47 | 0.07 |  |  |
|  | 0.5% | 1 | 24.75 | 32.05 | 7.30 | 0.90 | 0.91 | 1.00±0.4 |
|  |  | 2 | 24.77 | 32.17 | 7.40 | 1.00 |  |  |
|  |  | 3 | 24.82 | 32.05 | 7.23 | 0.83 |  |  |

**TABLE S8 Calculation of ΔΔCt Values from Inter-laboratory Collaborative Validation Data (GM Soybean ZH6106 at 0.1% Content)**

| Lab | Sample | | Reference gene Ct | Event Ct | ΔCt | ΔΔCt | Average ΔΔCt | Theoretical ΔΔCt |
| --- | --- | --- | --- | --- | --- | --- | --- | --- |
| Lab1 | QC（1%） | / | 22.08 | 31.80 | 9.72 | / | / | / |
|  | 0.1% | 1 | 22.21 | 35.28 | 13.08 | 3.36 | 3.18 | 3.32±0.4 |
|  |  | 2 | 22.15 | 35.09 | 12.94 | 3.22 |  |  |
|  |  | 3 | 22.16 | 34.85 | 12.69 | 2.97 |  |  |
| Lab2 | QC（1%） | / | 25.33 | 32.50 | 7.17 | / | / | / |
|  | 0.1% | 1 | 25.38 | 35.76 | 10.38 | 3.21 | 3.32 | 3.32±0.4 |
|  |  | 2 | 25.40 | 35.92 | 10.52 | 3.35 |  |  |
|  |  | 3 | 25.27 | 35.83 | 10.56 | 3.39 |  |  |
| Lab3 | QC（1%） | / | 24.80 | 32.82 | 8.02 | / | / | / |
|  | 0.1% | 1 | 24.76 | 36.16 | 11.40 | 3.38 | 3.30 | 3.32±0.4 |
|  |  | 2 | 24.77 | 36.04 | 11.27 | 3.26 |  |  |
|  |  | 3 | 24.64 | 35.91 | 11.27 | 3.26 |  |  |
| Lab4 | QC（1%） | / | 24.25 | 30.43 | 6.17 | / | / | / |
|  | 0.1% | 1 | 24.33 | 34.16 | 9.83 | 3.65 | 3.52 | 3.32±0.4 |
|  |  | 2 | 24.43 | 34.06 | 9.63 | 3.46 |  |  |
|  |  | 3 | 24.46 | 34.09 | 9.63 | 3.46 |  |  |
| Lab5 | QC（1%） | / | 24.05 | 30.78 | 6.73 | / | / | / |
|  | 0.1% | 1 | 24.09 | 34.18 | 10.09 | 3.35 | 3.39 | 3.32±0.4 |
|  |  | 2 | 24.11 | 34.35 | 10.24 | 3.51 |  |  |
|  |  | 3 | 24.17 | 34.20 | 10.03 | 3.30 |  |  |
| Lab6 | QC（1%） | / | 24.55 | 31.57 | 7.02 | / | / | / |
|  | 0.1% | 1 | 23.70 | 33.81 | 10.11 | 3.09 | 3.06 | 3.32±0.4 |
|  |  | 2 | 23.71 | 33.77 | 10.06 | 3.04 |  |  |
|  |  | 3 | 23.66 | 33.74 | 10.08 | 3.06 |  |  |
| Lab7 | QC（1%） | / | 21.60 | 28.34 | 6.74 | / | / | / |
|  | 0.1% | 1 | 21.49 | 31.80 | 10.31 | 3.57 | 3.59 | 3.32±0.4 |
|  |  | 2 | 21.48 | 31.78 | 10.30 | 3.57 |  |  |
|  |  | 3 | 21.48 | 31.85 | 10.37 | 3.63 |  |  |
| Lab8 | QC（1%） | / | 24.42 | 31.12 | 6.70 | / | / | / |
|  | 0.1% | 1 | 24.42 | 34.07 | 9.65 | 2.95 | 2.95 | 3.32±0.4 |
|  |  | 2 | 24.33 | 33.98 | 9.66 | 2.96 |  |  |
|  |  | 3 | 24.39 | 34.03 | 9.64 | 2.95 |  |  |

**TABLE S9 Calculation of ΔΔCt Values from Inter-laboratory Collaborative Validation Data (GM maize BFL4-2 at 5% and 2% Content)**

| Lab | Sample | | Reference gene Ct | Event Ct | ΔCt | ΔΔCt | Average ΔΔCt | Theoretical ΔΔCt |
| --- | --- | --- | --- | --- | --- | --- | --- | --- |
| Lab1 | QC（1%） | / | 25.67 | 32.71 | 7.04 | / | / | / |
|  | 5% | 1 | 25.63 | 30.30 | 4.67 | -2.37 | -2.33 | -2.32±0.4 |
|  |  | 2 | 25.57 | 30.31 | 4.74 | -2.30 |  |  |
|  |  | 3 | 25.48 | 30.21 | 4.73 | -2.31 |  |  |
|  | 2% | 1 | 25.48 | 31.80 | 6.32 | -0.72 | -0.81 | -1.00±0.4 |
|  |  | 2 | 25.43 | 31.63 | 6.20 | -0.84 |  |  |
|  |  | 3 | 25.49 | 31.65 | 6.16 | -0.88 |  |  |
| Lab2 | QC（1%） | / | 25.97 | 31.22 | 5.25 | / | / | / |
|  | 5% | 1 | 26.09 | 29.03 | 2.94 | -2.31 | -2.47 | -2.32±0.4 |
|  |  | 2 | 26.22 | 28.89 | 2.68 | -2.57 |  |  |
|  |  | 3 | 26.46 | 29.18 | 2.72 | -2.53 |  |  |
|  | 2% | 1 | 26.13 | 30.32 | 4.19 | -1.06 | -1.16 | -1.00±0.4 |
|  |  | 2 | 27.03 | 30.98 | 3.95 | -1.30 |  |  |
|  |  | 3 | 26.31 | 30.43 | 4.13 | -1.12 |  |  |
| Lab3 | QC（1%） | / | 27.67 | 32.85 | 5.18 | / | / | / |
|  | 5% | 1 | 27.55 | 30.48 | 2.93 | -2.25 | -2.19 | -2.32±0.4 |
|  |  | 2 | 27.60 | 30.62 | 3.03 | -2.15 |  |  |
|  |  | 3 | 27.62 | 30.63 | 3.01 | -2.17 |  |  |
|  | 2% | 1 | 27.50 | 31.71 | 4.21 | -0.97 | -0.87 | -1.00±0.4 |
|  |  | 2 | 27.52 | 31.86 | 4.34 | -0.84 |  |  |
|  |  | 3 | 27.58 | 31.95 | 4.37 | -0.81 |  |  |
| Lab4 | QC（1%） | / | 27.05 | 30.50 | 3.45 | / | / | / |
|  | 5% | 1 | 27.39 | 28.29 | 0.90 | -2.55 | -2.42 | -2.32±0.4 |
|  |  | 2 | 28.39 | 29.50 | 1.12 | -2.34 |  |  |
|  |  | 3 | 27.06 | 28.14 | 1.08 | -2.37 |  |  |
|  | 2% | 1 | 27.61 | 29.59 | 1.98 | -1.47 | -1.44 | -1.00±0.4 |
|  |  | 2 | 27.27 | 29.54 | 2.27 | -1.19 |  |  |
|  |  | 3 | 27.71 | 29.49 | 1.78 | -1.67 |  |  |
| Lab5 | QC（1%） | / | 25.34 | 31.97 | 6.64 | / | / | / |
|  | 5% | 1 | 25.33 | 29.66 | 4.33 | -2.31 | -2.25 | -2.32±0.4 |
|  |  | 2 | 25.20 | 29.64 | 4.44 | -2.19 |  |  |
|  |  | 3 | 25.23 | 29.60 | 4.38 | -2.26 |  |  |
|  | 2% | 1 | 25.14 | 30.84 | 5.70 | -0.94 | -0.74 | -1.00±0.4 |
|  |  | 2 | 24.79 | 30.81 | 6.02 | -0.62 |  |  |
|  |  | 3 | 24.94 | 30.93 | 5.98 | -0.65 |  |  |
| Lab6 | QC（1%） | / | 25.52 | 31.25 | 5.73 | / | / | / |
|  | 5% | 1 | 25.06 | 28.60 | 3.54 | -2.19 | -2.23 | -2.32±0.4 |
|  |  | 2 | 25.08 | 28.55 | 3.47 | -2.26 |  |  |
|  |  | 3 | 25.11 | 28.59 | 3.48 | -2.25 |  |  |
|  | 2% | 1 | 25.25 | 29.97 | 4.72 | -1.01 | -0.95 | -1.00±0.4 |
|  |  | 2 | 25.32 | 30.10 | 4.78 | -0.95 |  |  |
|  |  | 3 | 25.29 | 30.13 | 4.84 | -0.89 |  |  |
| Lab7 | QC（1%） | / | 22.78 | 30.14 | 7.35 | / | / | / |
|  | 5% | 1 | 22.91 | 27.98 | 5.07 | -2.28 | -2.27 | -2.32±0.4 |
|  |  | 2 | 22.98 | 27.99 | 5.01 | -2.34 |  |  |
|  |  | 3 | 22.91 | 28.08 | 5.17 | -2.18 |  |  |
|  | 2% | 1 | 22.66 | 29.05 | 6.40 | -0.96 | -0.97 | -1.00±0.4 |
|  |  | 2 | 22.70 | 29.11 | 6.41 | -0.94 |  |  |
|  |  | 3 | 22.70 | 29.05 | 6.35 | -1.01 |  |  |
| Lab8 | QC（1%） | / | 24.66 | 31.77 | 7.12 | / | / | / |
|  | 5% | 1 | 24.90 | 29.44 | 4.55 | -2.57 | -2.47 | -2.32±0.4 |
|  |  | 2 | 25.45 | 30.13 | 4.68 | -2.43 |  |  |
|  |  | 3 | 24.96 | 29.66 | 4.70 | -2.42 |  |  |
|  | 2% | 1 | 24.93 | 31.02 | 6.09 | -1.02 | -1.08 | -1.00±0.4 |
|  |  | 2 | 25.11 | 31.17 | 6.06 | -1.06 |  |  |
|  |  | 3 | 24.77 | 30.74 | 5.97 | -1.15 |  |  |

**TABLE S10 Calculation of ΔΔCt Values from Inter-laboratory Collaborative Validation Data (GM maize BFL4-2 at 1% and 0.5% Content)**

| Lab | Sample | | Reference gene Ct | Event Ct | ΔCt | ΔΔCt | Average ΔΔCt | Theoretical ΔΔCt |
| --- | --- | --- | --- | --- | --- | --- | --- | --- |
| Lab1 | QC（1%） | / | 24.99 | 32.66 | 7.67 | / | / | / |
|  | 1% | 1 | 25.33 | 32.54 | 7.21 | -0.45 | -0.22 | 0.00±0.4 |
|  |  | 2 | 25.09 | 32.62 | 7.52 | -0.14 |  |  |
|  |  | 3 | 25.08 | 32.70 | 7.62 | -0.05 |  |  |
|  | 0.5% | 1 | 24.91 | 33.66 | 8.75 | 1.09 | 1.03 | 1.00±0.4 |
|  |  | 2 | 25.17 | 33.80 | 8.63 | 0.97 |  |  |
|  |  | 3 | 25.08 | 33.78 | 8.70 | 1.04 |  |  |
| Lab2 | QC（1%） | / | 26.06 | 32.61 | 6.55 | / | / | / |
|  | 1% | 1 | 26.08 | 32.78 | 6.70 | 0.15 | 0.13 | 0.00±0.4 |
|  |  | 2 | 25.95 | 32.63 | 6.68 | 0.14 |  |  |
|  |  | 3 | 26.39 | 33.04 | 6.66 | 0.11 |  |  |
|  | 0.5% | 1 | 26.06 | 33.88 | 7.82 | 1.28 | 1.04 | 1.00±0.4 |
|  |  | 2 | 26.00 | 33.31 | 7.31 | 0.76 |  |  |
|  |  | 3 | 25.97 | 33.60 | 7.63 | 1.08 |  |  |
| Lab3 | QC（1%） | / | 27.68 | 32.82 | 5.14 | / | / | / |
|  | 1% | 1 | 27.65 | 32.88 | 5.23 | 0.09 | 0.09 | 0.00±0.4 |
|  |  | 2 | 27.63 | 32.81 | 5.18 | 0.04 |  |  |
|  |  | 3 | 27.70 | 32.98 | 5.28 | 0.14 |  |  |
|  | 0.5% | 1 | 27.71 | 34.11 | 6.40 | 1.26 | 1.25 | 1.00±0.4 |
|  |  | 2 | 27.49 | 33.89 | 6.39 | 1.25 |  |  |
|  |  | 3 | 27.78 | 34.16 | 6.38 | 1.24 |  |  |
| Lab4 | QC（1%） | / | 27.16 | 30.33 | 3.17 | / | / | / |
|  | 1% | 1 | 27.12 | 30.22 | 3.10 | -0.07 | 0.01 | 0.00±0.4 |
|  |  | 2 | 27.10 | 30.35 | 3.25 | 0.08 |  |  |
|  |  | 3 | 27.01 | 30.18 | 3.17 | 0.00 |  |  |
|  | 0.5% | 1 | 27.00 | 31.13 | 4.12 | 0.95 | 0.97 | 1.00±0.4 |
|  |  | 2 | 27.15 | 31.32 | 4.17 | 1.00 |  |  |
|  |  | 3 | 27.15 | 31.26 | 4.12 | 0.95 |  |  |
| Lab5 | QC（1%） | / | 25.38 | 31.85 | 6.47 | / | / | / |
|  | 1% | 1 | 25.67 | 31.87 | 6.19 | -0.28 | -0.03 | 0.00±0.4 |
|  |  | 2 | 25.06 | 31.77 | 6.71 | 0.24 |  |  |
|  |  | 3 | 25.41 | 31.82 | 6.42 | -0.05 |  |  |
|  | 0.5% | 1 | 25.27 | 32.54 | 7.27 | 0.80 | 0.92 | 1.00±0.4 |
|  |  | 2 | 25.17 | 32.60 | 7.43 | 0.96 |  |  |
|  |  | 3 | 25.18 | 32.65 | 7.46 | 0.99 |  |  |
| Lab6 | QC（1%） | / | 25.41 | 30.93 | 5.52 | / | / | / |
|  | 1% | 1 | 25.62 | 31.22 | 5.60 | 0.08 | 0.01 | 0.00±0.4 |
|  |  | 2 | 25.42 | 30.90 | 5.48 | -0.05 |  |  |
|  |  | 3 | 25.54 | 31.05 | 5.52 | -0.01 |  |  |
|  | 0.5% | 1 | 25.41 | 31.92 | 6.51 | 0.99 | 1.01 | 1.00±0.4 |
|  |  | 2 | 25.28 | 31.85 | 6.57 | 1.05 |  |  |
|  |  | 3 | 25.42 | 31.92 | 6.50 | 0.98 |  |  |
| Lab7 | QC（1%） | / | 22.77 | 30.12 | 7.34 | / | / | / |
|  | 1% | 1 | 22.91 | 30.39 | 7.47 | 0.13 | 0.07 | 0.00±0.4 |
|  |  | 2 | 22.88 | 30.25 | 7.36 | 0.02 |  |  |
|  |  | 3 | 22.96 | 30.37 | 7.41 | 0.07 |  |  |
|  | 0.5% | 1 | 22.80 | 31.32 | 8.51 | 1.17 | 1.10 | 1.00±0.4 |
|  |  | 2 | 22.78 | 31.18 | 8.40 | 1.05 |  |  |
|  |  | 3 | 22.76 | 31.18 | 8.41 | 1.07 |  |  |
| Lab8 | QC（1%） | / | 24.90 | 31.66 | 6.77 | / | / | / |
|  | 1% | 1 | 25.08 | 31.97 | 6.89 | 0.13 | 0.06 | 0.00±0.4 |
|  |  | 2 | 25.05 | 31.78 | 6.74 | -0.03 |  |  |
|  |  | 3 | 25.02 | 31.87 | 6.84 | 0.08 |  |  |
|  | 0.5% | 1 | 25.32 | 33.26 | 7.95 | 1.18 | 1.10 | 1.00±0.4 |
|  |  | 2 | 24.95 | 32.79 | 7.84 | 1.07 |  |  |
|  |  | 3 | 24.74 | 32.55 | 7.81 | 1.04 |  |  |

**TABLE S11 Calculation of ΔΔCt Values from Inter-laboratory Collaborative Validation Data (GM maize BFL4-2 at 0.1% Content)**

| Lab | Sample | | Reference gene Ct | Event Ct | ΔCt | ΔΔCt | Average ΔΔCt | Theoretical ΔΔCt |
| --- | --- | --- | --- | --- | --- | --- | --- | --- |
| Lab1 | QC（1%） | / | 25.92 | 32.38 | 6.46 | / | / | / |
|  | 0.1% | 1 | 25.52 | 35.03 | 9.51 | 3.05 | 3.37 | 3.32±0.4 |
|  |  | 2 | 25.41 | 35.44 | 10.03 | 3.57 |  |  |
|  |  | 3 | 25.34 | 35.29 | 9.94 | 3.48 |  |  |
| Lab2 | QC（1%） | / | 25.99 | 31.16 | 5.16 | / | / | / |
|  | 0.1% | 1 | 25.67 | 33.89 | 8.22 | 3.06 | 3.26 | 3.32±0.4 |
|  |  | 2 | 25.81 | 34.44 | 8.63 | 3.47 |  |  |
|  |  | 3 | 25.82 | 34.24 | 8.42 | 3.26 |  |  |
| Lab3 | QC（1%） | / | 27.69 | 32.76 | 5.07 | / | / | / |
|  | 0.1% | 1 | 27.13 | 35.59 | 8.46 | 3.39 | 3.46 | 3.32±0.4 |
|  |  | 2 | 27.12 | 35.69 | 8.57 | 3.50 |  |  |
|  |  | 3 | 27.20 | 35.77 | 8.57 | 3.50 |  |  |
| Lab4 | QC（1%） | / | 24.72 | 30.63 | 5.90 | / | / | / |
|  | 0.1% | 1 | 24.18 | 33.47 | 9.29 | 3.39 | 3.17 | 3.32±0.4 |
|  |  | 2 | 24.14 | 32.96 | 8.82 | 2.91 |  |  |
|  |  | 3 | 24.33 | 33.44 | 9.11 | 3.20 |  |  |
| Lab5 | QC（1%） | / | 25.42 | 31.80 | 6.38 | / | / | / |
|  | 0.1% | 1 | 24.95 | 35.00 | 10.05 | 3.67 | 3.40 | 3.32±0.4 |
|  |  | 2 | 25.05 | 34.91 | 9.86 | 3.49 |  |  |
|  |  | 3 | 25.09 | 34.51 | 9.42 | 3.05 |  |  |
| Lab6 | QC（1%） | / | 25.61 | 30.16 | 4.55 | / | / | / |
|  | 0.1% | 1 | 24.77 | 32.29 | 7.51 | 2.96 | 3.06 | 3.32±0.4 |
|  |  | 2 | 24.69 | 32.42 | 7.73 | 3.18 |  |  |
|  |  | 3 | 24.77 | 32.35 | 7.58 | 3.03 |  |  |
| Lab7 | QC（1%） | / | 22.61 | 29.97 | 7.36 | / | / | / |
|  | 0.1% | 1 | 22.35 | 33.13 | 10.78 | 3.42 | 3.33 | 3.32±0.4 |
|  |  | 2 | 22.35 | 33.03 | 10.68 | 3.32 |  |  |
|  |  | 3 | 22.45 | 33.05 | 10.60 | 3.24 |  |  |
| Lab8 | QC（1%） | / | 24.85 | 31.86 | 7.01 | / | / | / |
|  | 0.1% | 1 | 24.40 | 34.50 | 10.10 | 3.09 | 3.14 | 3.32±0.4 |
|  |  | 2 | 24.49 | 34.79 | 10.30 | 3.28 |  |  |
|  |  | 3 | 24.49 | 34.55 | 10.07 | 3.06 |  |  |

**TABLE S12 Calculation of ΔΔCt Values from Inter-laboratory Collaborative Validation Data (GM maize BBL2-2 at 5% and 3% Content)**

| Lab | Sample | | Reference gene Ct | Event Ct | ΔCt | ΔΔCt | Average ΔΔCt | Theoretical ΔΔCt |
| --- | --- | --- | --- | --- | --- | --- | --- | --- |
| Lab1 | QC（3%） | / | 25.38 | 30.52 | 5.14 | / | / | / |
|  | 5% | 1 | 25.49 | 29.80 | 4.30 | -0.84 | -0.87 | -0.74±0.4 |
|  |  | 2 | 25.55 | 29.81 | 4.27 | -0.87 |  |  |
|  |  | 3 | 25.46 | 29.70 | 4.24 | -0.90 |  |  |
|  | 3% | 1 | 25.47 | 30.61 | 5.14 | 0.00 | -0.02 | 0.00±0.4 |
|  |  | 2 | 25.52 | 30.63 | 5.12 | -0.02 |  |  |
|  |  | 3 | 25.39 | 30.51 | 5.12 | -0.02 |  |  |
| Lab2 | QC（3%） | / | 25.30 | 30.39 | 5.09 | / | / | / |
|  | 5% | 1 | 25.40 | 29.75 | 4.35 | -0.74 | -0.74 | -0.74±0.4 |
|  |  | 2 | 25.60 | 29.97 | 4.36 | -0.73 |  |  |
|  |  | 3 | 25.36 | 29.71 | 4.35 | -0.74 |  |  |
|  | 3% | 1 | 25.43 | 30.55 | 5.13 | 0.04 | 0.05 | 0.00±0.4 |
|  |  | 2 | 25.69 | 30.81 | 5.13 | 0.04 |  |  |
|  |  | 3 | 25.43 | 30.60 | 5.16 | 0.07 |  |  |
| Lab3 | QC（3%） | / | 26.56 | 33.71 | 7.16 | / | / | / |
|  | 5% | 1 | 26.44 | 32.81 | 6.37 | -0.79 | -0.78 | -0.74±0.4 |
|  |  | 2 | 26.42 | 32.82 | 6.40 | -0.75 |  |  |
|  |  | 3 | 26.41 | 32.77 | 6.36 | -0.80 |  |  |
|  | 3% | 1 | 26.43 | 33.66 | 7.23 | 0.08 | 0.02 | 0.00±0.4 |
|  |  | 2 | 26.43 | 33.54 | 7.10 | -0.05 |  |  |
|  |  | 3 | 26.50 | 33.70 | 7.20 | 0.05 |  |  |
| Lab4 | QC（3%） | / | 25.02 | 30.04 | 5.02 | / | / | / |
|  | 5% | 1 | 25.57 | 29.54 | 3.96 | -1.06 | -1.03 | -0.74±0.4 |
|  |  | 2 | 25.29 | 29.29 | 4.00 | -1.03 |  |  |
|  |  | 3 | 25.03 | 29.04 | 4.01 | -1.01 |  |  |
|  | 3% | 1 | 25.02 | 29.98 | 4.96 | -0.07 | 0.04 | 0.00±0.4 |
|  |  | 2 | 24.91 | 29.92 | 5.01 | -0.01 |  |  |
|  |  | 3 | 24.82 | 30.05 | 5.23 | 0.21 |  |  |
| Lab5 | QC（3%） | / | 25.21 | 28.91 | 3.71 | / | / | / |
|  | 5% | 1 | 25.23 | 28.67 | 3.44 | -0.27 | -0.38 | -0.74±0.4 |
|  |  | 2 | 25.22 | 28.53 | 3.31 | -0.40 |  |  |
|  |  | 3 | 25.35 | 28.59 | 3.25 | -0.46 |  |  |
|  | 3% | 1 | 25.25 | 29.21 | 3.96 | 0.25 | 0.30 | 0.00±0.4 |
|  |  | 2 | 25.68 | 29.66 | 3.98 | 0.27 |  |  |
|  |  | 3 | 25.24 | 29.33 | 4.09 | 0.39 |  |  |
| Lab6 | QC（3%） | / | 25.90 | 30.53 | 4.63 | / | / | / |
|  | 5% | 1 | 25.78 | 29.74 | 3.96 | -0.67 | -0.69 | -0.74±0.4 |
|  |  | 2 | 25.98 | 29.85 | 3.87 | -0.76 |  |  |
|  |  | 3 | 25.93 | 29.92 | 3.99 | -0.64 |  |  |
|  | 3% | 1 | 25.88 | 30.58 | 4.70 | 0.07 | 0.05 | 0.00±0.4 |
|  |  | 2 | 25.86 | 30.50 | 4.64 | 0.01 |  |  |
|  |  | 3 | 25.92 | 30.61 | 4.69 | 0.07 |  |  |
| Lab7 | QC（3%） | / | 23.20 | 28.35 | 5.15 | / | / | / |
|  | 5% | 1 | 23.15 | 27.61 | 4.45 | -0.69 | -0.74 | -0.74±0.4 |
|  |  | 2 | 23.24 | 27.59 | 4.35 | -0.79 |  |  |
|  |  | 3 | 23.29 | 27.72 | 4.43 | -0.72 |  |  |
|  | 3% | 1 | 23.25 | 28.54 | 5.29 | 0.14 | 0.11 | 0.00±0.4 |
|  |  | 2 | 23.14 | 28.34 | 5.20 | 0.06 |  |  |
|  |  | 3 | 23.28 | 28.57 | 5.29 | 0.14 |  |  |
| Lab8 | QC（3%） | / | 24.93 | 30.20 | 5.27 | / | / | / |
|  | 5% | 1 | 24.92 | 29.65 | 4.73 | -0.54 | -0.51 | -0.74±0.4 |
|  |  | 2 | 24.89 | 29.68 | 4.79 | -0.48 |  |  |
|  |  | 3 | 24.92 | 29.67 | 4.76 | -0.52 |  |  |
|  | 3% | 1 | 24.90 | 30.52 | 5.62 | 0.35 | 0.30 | 0.00±0.4 |
|  |  | 2 | 24.68 | 30.29 | 5.61 | 0.34 |  |  |
|  |  | 3 | 25.00 | 30.50 | 5.50 | 0.23 |  |  |

**TABLE S13 Calculation of ΔΔCt Values from Inter-laboratory Collaborative Validation Data (GM maize BBL2-2 at 1% and 0.5% Content)**

| Lab | Sample | | Reference gene Ct | Event Ct | ΔCt | ΔΔCt | Average ΔΔCt | Theoretical ΔΔCt |
| --- | --- | --- | --- | --- | --- | --- | --- | --- |
| Lab1 | QC（3%） | / | 25.49 | 30.63 | 5.14 | / | / | / |
|  | 1% | 1 | 25.60 | 32.22 | 6.62 | 1.48 | 1.53 | 1.58±0.4 |
|  |  | 2 | 25.57 | 32.29 | 6.72 | 1.58 |  |  |
|  |  | 3 | 25.62 | 32.31 | 6.69 | 1.55 |  |  |
|  | 0.5% | 1 | 25.54 | 33.16 | 7.62 | 2.48 | 2.50 | 2.58±0.4 |
|  |  | 2 | 25.49 | 33.12 | 7.63 | 2.49 |  |  |
|  |  | 3 | 25.55 | 33.21 | 7.66 | 2.52 |  |  |
| Lab2 | QC（3%） | / | 25.34 | 30.94 | 5.60 | / | / | / |
|  | 1% | 1 | 25.43 | 32.57 | 7.14 | 1.54 | 1.59 | 1.58±0.4 |
|  |  | 2 | 25.62 | 32.74 | 7.12 | 1.53 |  |  |
|  |  | 3 | 25.37 | 32.67 | 7.30 | 1.70 |  |  |
|  | 0.5% | 1 | 25.42 | 33.41 | 7.99 | 2.39 | 2.40 | 2.58±0.4 |
|  |  | 2 | 25.66 | 33.61 | 7.95 | 2.36 |  |  |
|  |  | 3 | 25.38 | 33.44 | 8.05 | 2.46 |  |  |
| Lab3 | QC（3%） | / | 26.54 | 33.71 | 7.17 | / | / | / |
|  | 1% | 1 | 26.31 | 35.22 | 8.91 | 1.74 | 1.77 | 1.58±0.4 |
|  |  | 2 | 26.34 | 35.31 | 8.98 | 1.81 |  |  |
|  |  | 3 | 26.34 | 35.27 | 8.93 | 1.76 |  |  |
|  | 0.5% | 1 | 26.31 | 36.17 | 9.86 | 2.69 | 2.84 | 2.58±0.4 |
|  |  | 2 | 26.31 | 36.39 | 10.09 | 2.92 |  |  |
|  |  | 3 | 26.36 | 36.43 | 10.07 | 2.90 |  |  |
| Lab4 | QC（3%） | / | 25.04 | 29.68 | 4.64 | / | / | / |
|  | 1% | 1 | 25.60 | 31.43 | 5.83 | 1.18 | 1.37 | 1.58±0.4 |
|  |  | 2 | 25.23 | 31.27 | 6.04 | 1.40 |  |  |
|  |  | 3 | 25.14 | 31.30 | 6.16 | 1.52 |  |  |
|  | 0.5% | 1 | 25.01 | 32.05 | 7.04 | 2.40 | 2.43 | 2.58±0.4 |
|  |  | 2 | 24.82 | 31.94 | 7.12 | 2.47 |  |  |
|  |  | 3 | 24.98 | 32.04 | 7.06 | 2.42 |  |  |
| Lab5 | QC（3%） | / | 25.56 | 28.13 | 2.57 | / | / | / |
|  | 1% | 1 | 25.47 | 30.12 | 4.65 | 2.08 | 1.98 | 1.58±0.4 |
|  |  | 2 | 25.44 | 30.03 | 4.59 | 2.02 |  |  |
|  |  | 3 | 25.48 | 29.90 | 4.42 | 1.85 |  |  |
|  | 0.5% | 1 | 25.43 | 30.74 | 5.31 | 2.74 | 2.84 | 2.58±0.4 |
|  |  | 2 | 25.41 | 30.89 | 5.48 | 2.91 |  |  |
|  |  | 3 | 25.46 | 30.90 | 5.44 | 2.87 |  |  |
| Lab6 | QC（3%） | / | 25.89 | 30.46 | 4.57 | / | / | / |
|  | 1% | 1 | 25.79 | 31.90 | 6.12 | 1.54 | 1.47 | 1.58±0.4 |
|  |  | 2 | 25.87 | 31.87 | 6.00 | 1.42 |  |  |
|  |  | 3 | 25.88 | 31.88 | 6.00 | 1.43 |  |  |
|  | 0.5% | 1 | 25.93 | 32.99 | 7.05 | 2.48 | 2.44 | 2.58±0.4 |
|  |  | 2 | 25.88 | 32.83 | 6.95 | 2.38 |  |  |
|  |  | 3 | 25.87 | 32.90 | 7.03 | 2.45 |  |  |
| Lab7 | QC（3%） | / | 23.18 | 28.35 | 5.17 | / | / | / |
|  | 1% | 1 | 23.12 | 29.84 | 6.72 | 1.56 | 1.64 | 1.58±0.4 |
|  |  | 2 | 23.14 | 30.02 | 6.88 | 1.72 |  |  |
|  |  | 3 | 23.15 | 29.97 | 6.82 | 1.66 |  |  |
|  | 0.5% | 1 | 23.13 | 31.02 | 7.89 | 2.73 | 2.62 | 2.58±0.4 |
|  |  | 2 | 23.12 | 30.80 | 7.68 | 2.52 |  |  |
|  |  | 3 | 23.17 | 30.95 | 7.78 | 2.61 |  |  |
| Lab8 | QC（3%） | / | 24.64 | 30.14 | 5.51 | / | / | / |
|  | 1% | 1 | 24.72 | 32.13 | 7.42 | 1.91 | 1.91 | 1.58±0.4 |
|  |  | 2 | 24.65 | 32.04 | 7.40 | 1.89 |  |  |
|  |  | 3 | 24.59 | 32.03 | 7.44 | 1.93 |  |  |
|  | 0.5% | 1 | 24.48 | 32.70 | 8.23 | 2.72 | 2.82 | 2.58±0.4 |
|  |  | 2 | 24.61 | 33.02 | 8.41 | 2.91 |  |  |
|  |  | 3 | 24.73 | 33.06 | 8.34 | 2.83 |  |  |

**TABLE S14 Calculation of ΔΔCt Values from Inter-laboratory Collaborative Validation Data (GM maize BBL2-2 at 0.1% Content)**

| Lab | Sample | | Reference gene Ct | Event Ct | ΔCt | ΔΔCt | Average ΔΔCt | Theoretical ΔΔCt |
| --- | --- | --- | --- | --- | --- | --- | --- | --- |
| Lab1 | QC（3%） | / | 25.52 | 30.52 | 5.01 | / | / | / |
|  | 0.1% | 1 | 25.58 | 35.30 | 9.71 | 4.71 | 4.86 | 4.91±0.4 |
|  |  | 2 | 25.57 | 35.62 | 10.05 | 5.04 |  |  |
|  |  | 3 | 25.47 | 35.32 | 9.84 | 4.84 |  |  |
| Lab2 | QC（3%） | / | 25.38 | 30.53 | 5.15 | / | / | / |
|  | 0.1% | 1 | 25.35 | 35.42 | 10.06 | 4.92 | 4.79 | 4.91±0.4 |
|  |  | 2 | 25.53 | 35.46 | 9.93 | 4.78 |  |  |
|  |  | 3 | 25.37 | 35.20 | 9.83 | 4.69 |  |  |
| Lab3 | QC（3%） | / | 26.58 | 33.72 | 7.14 | / | / | / |
|  | 0.1% | 1 | 26.51 | 38.81 | 12.30 | 5.15 | 5.19 | 4.91±0.4 |
|  |  | 2 | 26.50 | 38.93 | 12.43 | 5.28 |  |  |
|  |  | 3 | 26.52 | 38.80 | 12.28 | 5.14 |  |  |
| Lab4 | QC（3%） | / | 25.12 | 29.89 | 4.78 | / | / | / |
|  | 0.1% | 1 | 25.52 | 34.26 | 8.73 | 3.96 | 4.13 | 4.91±0.4 |
|  |  | 2 | 25.24 | 34.20 | 8.97 | 4.19 |  |  |
|  |  | 3 | 25.32 | 34.34 | 9.02 | 4.25 |  |  |
| Lab5 | QC（3%） | / | 25.49 | 27.95 | 2.46 | / | / | / |
|  | 0.1% | 1 | 25.45 | 33.17 | 7.72 | 5.25 | 5.00 | 4.91±0.4 |
|  |  | 2 | 25.68 | 33.03 | 7.35 | 4.89 |  |  |
|  |  | 3 | 25.50 | 32.81 | 7.31 | 4.85 |  |  |
| Lab6 | QC（3%） | / | 24.47 | 29.01 | 4.54 | / | / | / |
|  | 0.1% | 1 | 24.22 | 33.55 | 9.33 | 4.79 | 4.71 | 4.91±0.4 |
|  |  | 2 | 24.41 | 33.63 | 9.22 | 4.68 |  |  |
|  |  | 3 | 24.40 | 33.60 | 9.20 | 4.66 |  |  |
| Lab7 | QC（3%） | / | 23.31 | 28.49 | 5.18 | / | / | / |
|  | 0.1% | 1 | 22.97 | 32.71 | 9.74 | 4.56 | 4.56 | 4.91±0.4 |
|  |  | 2 | 23.50 | 33.28 | 9.78 | 4.60 |  |  |
|  |  | 3 | 23.09 | 32.79 | 9.70 | 4.52 |  |  |
| Lab8 | QC（3%） | / | 24.98 | 30.20 | 5.23 | / | / | / |
|  | 0.1% | 1 | 25.01 | 35.45 | 10.44 | 5.21 | 5.14 | 4.91±0.4 |
|  |  | 2 | 24.88 | 34.96 | 10.08 | 4.85 |  |  |
|  |  | 3 | 25.03 | 35.62 | 10.60 | 5.37 |  |  |
